# Pairing Electrocatalytic Reduction and Oxidation of Biomass-Derived 5-Hydroxymethylfurfural into Highly Value-Added Chemicals

**DOI:** 10.1021/jacsau.4c01135

**Published:** 2025-01-02

**Authors:** Man Zhang, Zhikeng Zheng, Xiaodie Zhang, Zhiwei Jiang, Xue Yong, Ke Li, Xin Tu, Kai Yan

**Affiliations:** † Guangdong Provincial Key Laboratory of Environmental Pollution Control and Remediation Technology, School of Environmental Science and Engineering, Sun Yat-sen University, Guangzhou 510275, China; ‡ College of Chemistry and Environment, Southwest University for Nationalities, Chengdu 610207, China; § Department of Electrical Engineering and Electronics, University of Liverpool, Liverpool L69 3GJ, U.K.

**Keywords:** pairing electrocatalytic reduction and oxidation, biomass-derived products, 5-hydroxymethylfurfural, kinetic isotope effects, universality

## Abstract

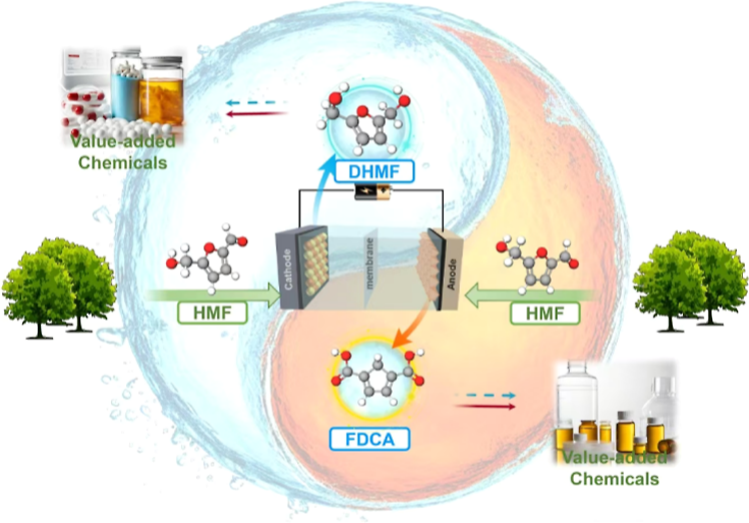

Simultaneous electrocatalytic reduction and oxidation of 5-hydroxymethylfurfural (HMF) is crucial for biomass refineries. Herein, we report the unprecedentedly high efficiency of the nearly complete conversion of biomass-derived HMF to value-added products, achieving >95% selectivity at −0.4 V vs RHE by pairing electrocatalytic reduction and oxidation (PERO) reactions in a single electrochemical cell. At the cathode, we achieved 99% conversion of HMF to 2,5-dihydroxymethylfuran (DHMF) in ∼99% yield under mild conditions using a PtRu alloy. At the anode, we observed 99% conversion of HMF, nearly perfect selectivity for the oxidative product 2,5-furandicarboxylic acid (FDCA), and 100% Faradaic efficiency on a NiCo(OOH)_*x*_ nanosheets electrode. The kinetic isotope effect demonstrated that the rate-controlled step was a proton-independent electron transfer process, with minimal impact from substrate concentration variations. After assembling the synchronous reaction cell, the PERO of HMF generated high yields of DHMF (94%) and FDCA (86%), achieving a combined electron efficiency of 131%, nearly doubling the performance of uncoupled cells. This superior performance was attributed to the efficient generation of H* on the PtRu alloy for reduction, alongside the OH* active sites on the NiCo(OOH)_*x*_ nanosheets electrode for oxidation. This research provides a promising strategy for the sustainable electrocatalytic upgrading of biomass-derived chemicals.

## Introduction

In comparison to traditional reduction and oxidation technologies, electrochemical biomass conversion has several pivotal advantages.^1−3^ First, the reactions can be conducted under ambient temperature and pressure conditions.^4^ Second, transformation reactions are driven by an applied potential, obviating the requirement for chemical reductants/oxidants as well as the imposition of high-pressure hydrogen or oxygen atmospheres.^2^ Third, electrochemical reduction or oxidation reactions can be strategically paired with simultaneous and advantageous half-reactions.^4^ An illustrative example is the oxidation of biomass-derived 5-hydroxymethylfurfural (HMF) to produce 2,5-furandicarboxylic acid (FDCA), which can be coupled with the reduction of water to hydrogen.^5,6^ This simultaneous occurrence of both reactions results in the generation of valuable products, thereby significantly increasing the overall efficiency and economic value of the process.

Electrochemical reduction has become an appealing and promising channel for the reductive transformation of HMF, particularly due to its operation under mild conditions, such as ambient temperature and pressure, utilizing water as the hydrogen source.^7,8^ This approach has been successfully applied to the conversion of HMF to 2,5-dihydroxymethylfuran (DHMF), achieving notable yields and high Faradaic efficiency (FE).^9−13^ Selecting catalysts based on previous research and experience and subsequently designing catalysts suitable for specific reduction reactions are crucial because they can inspire the exploration of various novel reaction systems.^14−16^ In electrocatalytic reduction systems, the formation of adsorbed hydrogen on metal (M) electrodes is a crucial factor influencing the electrochemical reduction process, determining the outcomes of various reduction reactions. A typical example is the volcano-type relationship between the hydrogen evolution reaction (HER) activity and the strength of the metal–hydrogen (M–H) bond. Optimal catalysts for the HER exhibit intermediate M–H bond strength, as illustrated by the volcano plot where the HER activity is plotted against the M–H bond strength (Figure S1).^17−19^ Metals with insufficient M–H bond strength fail to accumulate enough adsorbed hydrogen on their surfaces for effective hydrogen generation. Conversely, the hydrogen atoms are bonded too firmly when the M–H bond is excessively strong, which prevents them from being released as molecular hydrogen. Metals such as Pt and Ru, positioned near the optimum center of the volcano plot, demonstrate an ideal balance: strong enough to sustain significant hydrogen adsorption yet weak enough to facilitate the release of hydrogen gas, thereby optimizing HER activity. This concept of optimal M–H bond strength provides insights into the requirements for catalyst design in the electrochemical reduction of HMF. Specifically, the mechanism by which an optimum M–H bond strength can enhance HMF conversion needs to be thoroughly investigated, as the balance of hydrogen adsorption and release underlies both HER and HMF reactivity. Despite the effectiveness of noble metals such as Pt and Ru, which conform closely to this optimal bond strength range, their application can lead to the formation of byproducts. Therefore, the development of binary catalysts has emerged as a pivotal strategy for enhancing catalytic performance.^20−22^

In electrocatalytic reduction, H* is typically formed at the cathode and used to hydrogenate unsaturated substrates.^23^ At the same time, an oxidation reaction takes place at the anode, often producing low-value products such as oxygen, which suffer from high overpotentials due to four electron transfers and slow reaction kinetics. To address this, recent research has focused on coupled electrosynthesis, which aims to produce valuable products at the anode.^4,24,25^ The electrocatalytic oxidation mechanism of HMF was clarified on hydroxide, and an FDCA selectivity of 99% was demonstrated.^26−28^ At present, combining half-reactions with the HER has been strategically employed to maximize the generation of valuable products through electrocatalysis, thereby improving the energy efficiency of the process. Several such coupled electrochemical reactions have been successfully executed.^9^ However, the application of coupled half-reactions has a narrow scope and is significantly underutilized by the broader synthesis community.^29^ In particular, there is a lack of research on the coupled reaction of electrocatalytic reduction and oxidation of biomass-derived platform chemicals.

**Figure 1:**
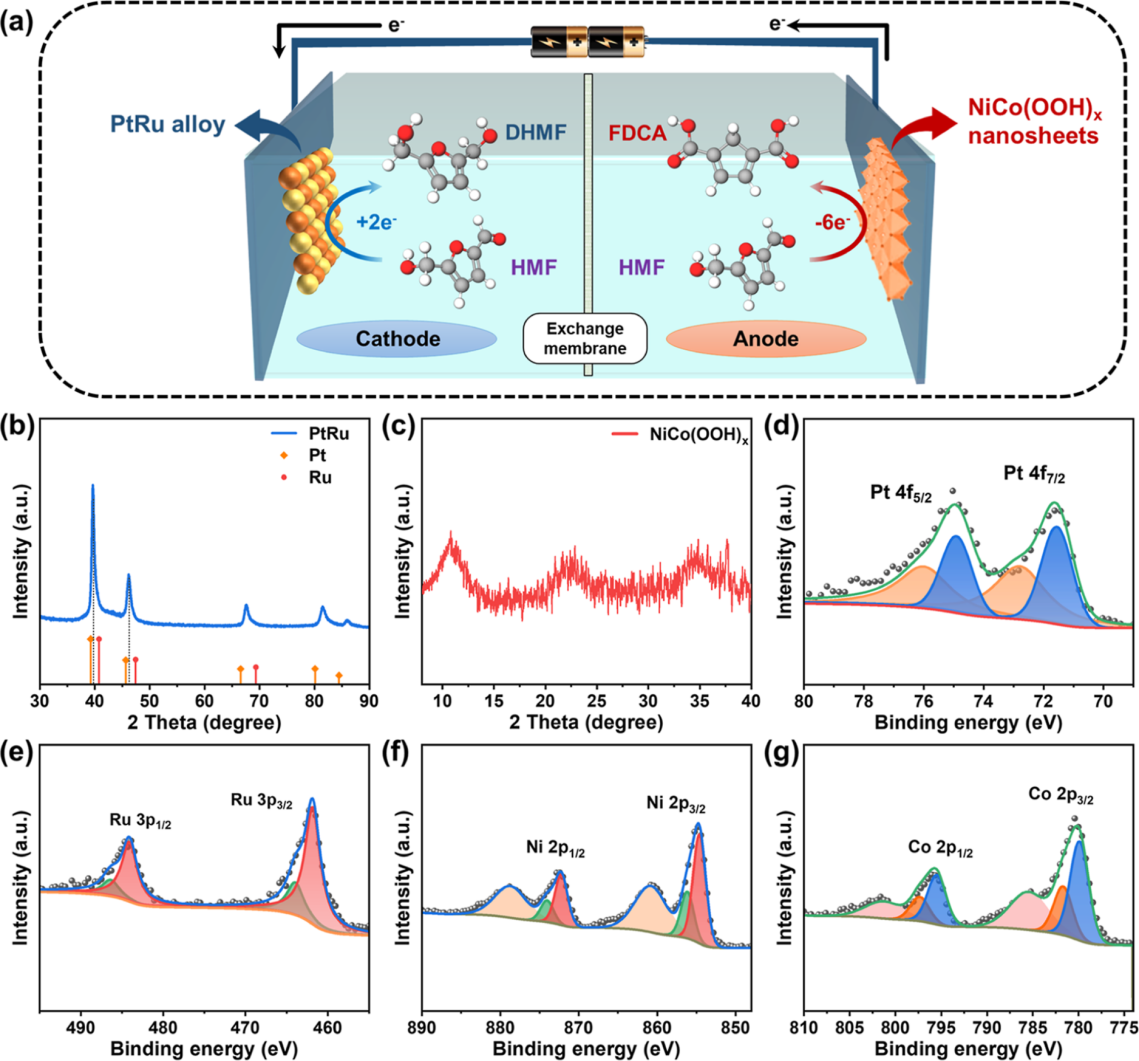
(a) Coupled electrolyzer for the electrocatalytic reduction of HMF to DHMF and the oxidation of HMF to FDCA. XRD patterns of (b) the PtRu alloy and (c) NiCo(OOH)_*x*_ nanosheets. XPS spectra of (d) Pt and (e) Ru in the PtRu alloy. XPS spectra of (f) Ni and (g) Co in NiCo(OOH)_*x*_ nanosheets.

In this study, we successfully constructed a high-efficiency pairing system for HMF reduction and oxidation in one assembled cell. We first carried out a comparative analysis of the voltammetric behavior and constant potential HMF reduction of the PtRu alloy to discern any patterns in how these metals influence the selectivity for HMF reduction. In addition, different from the methods used for monopolar reduction or oxidation reported in the literature, a coupled electrolyzer was constructed by combining the electrocatalytic reduction of HMF to DHMF and the oxidation of HMF to FDCA (Figure 1a). Using water as the source for hydrogen and oxygen, we achieved nearly perfect conversion and over 90% selectivity on both sides. The FE of the coupled system exceeded 130%, even after five runs. The high efficiency of the simultaneous reduction and oxidation of HMF exhibited by the electrocatalytic system was primarily due to the generation of H* on the PtRu alloy and OH* on the NiCo(OOH)_*x*_ nanosheets, as confirmed by in situ electron paramagnetic resonance (EPR), electrochemical impedance spectroscopy (EIS), and kinetic isotope effects (KIE). This pairing system would significantly improve the efficiency of biomass refining.

## Results and Discussion

### Structural Characterization of the Cathode and Anode

PtRu alloy catalysts were directly prepared using a solvent-free microwave-assisted strategy with a microwave power of 1300 W, which is a simple and swift synthesis process. The as-synthesized PtRu alloy was then used as a cathode for the electrocatalytic reduction of HMF. The crystallographic structure and associated diffraction peaks of the synthesized PtRu alloy were characterized using X-ray diffraction (XRD), and the results are shown in Figure 1b. The XRD patterns revealed five prominent peaks at 2θ angles of 39.7°, 46.2°, 67.5°, 81.2°, and 85.7°. The peaks corresponded well to the (111), (200), (220), (311), and (222) planes of a face-centered cubic structure.^30^ The positions of the characteristic peaks for the PtRu alloy were located between the standard 2θ peak positions of pure Pt (PDF no. 88-2343) and pure Ru (PDF no. 88–2333). This intermediate positioning suggested that the smaller Ru atoms were incorporated into the Pt lattice, leading to the formation of a PtRu alloy. This was particularly evident for the (111) plane, which exhibited a calculated interplanar spacing of 0.225 nm. The sharpness of these peaks was indicative of large grain sizes and a high degree of crystallinity, which implied the enhanced atomic order. In Figure 1c, the diffractogram of the NiCo(OOH)_*x*_ nanosheets shows characteristic hydroxide peaks at 11.1°, 22.0°, 33.5°, and 38.8°. Such well-defined crystallographic features were critical for influencing the electrocatalytic activity and durability.

X-ray photoelectron spectroscopy (XPS) was subsequently utilized to characterize the surface chemical states of the as-synthesized PtRu alloy. The high-resolution XPS spectrum of Pt 4f for the PtRu alloy, presented in Figure 1d, exhibited a detailed delineation into four distinct peaks. A pair of principal peaks attributable to Pt in its metallic state (Pt^0^) was observed at binding energies of 71.6 and 74.9 eV. The Ru 3p spectrum, shown in Figure 1e, featured binding energies at 461.9 and 464.1 eV, which were mainly attributed to the metallic state (Ru^0^). For the NiCo(OOH)_*x*_ nanosheets, high-resolution XPS spectra of Ni and Co were obtained. Figure 1f shows that the peaks at 872.5 and 854.7 eV correspond to the Ni 2p_1/2_ and Ni 2p_3/2_ signals, respectively.^31,32^ Two spin–orbit doublets of 795.6 and 780.2 eV were assigned to Co 2p_1/2_ and Co 2p_3/2_, respectively (Figure 1g).^33^

**Figure 2:**
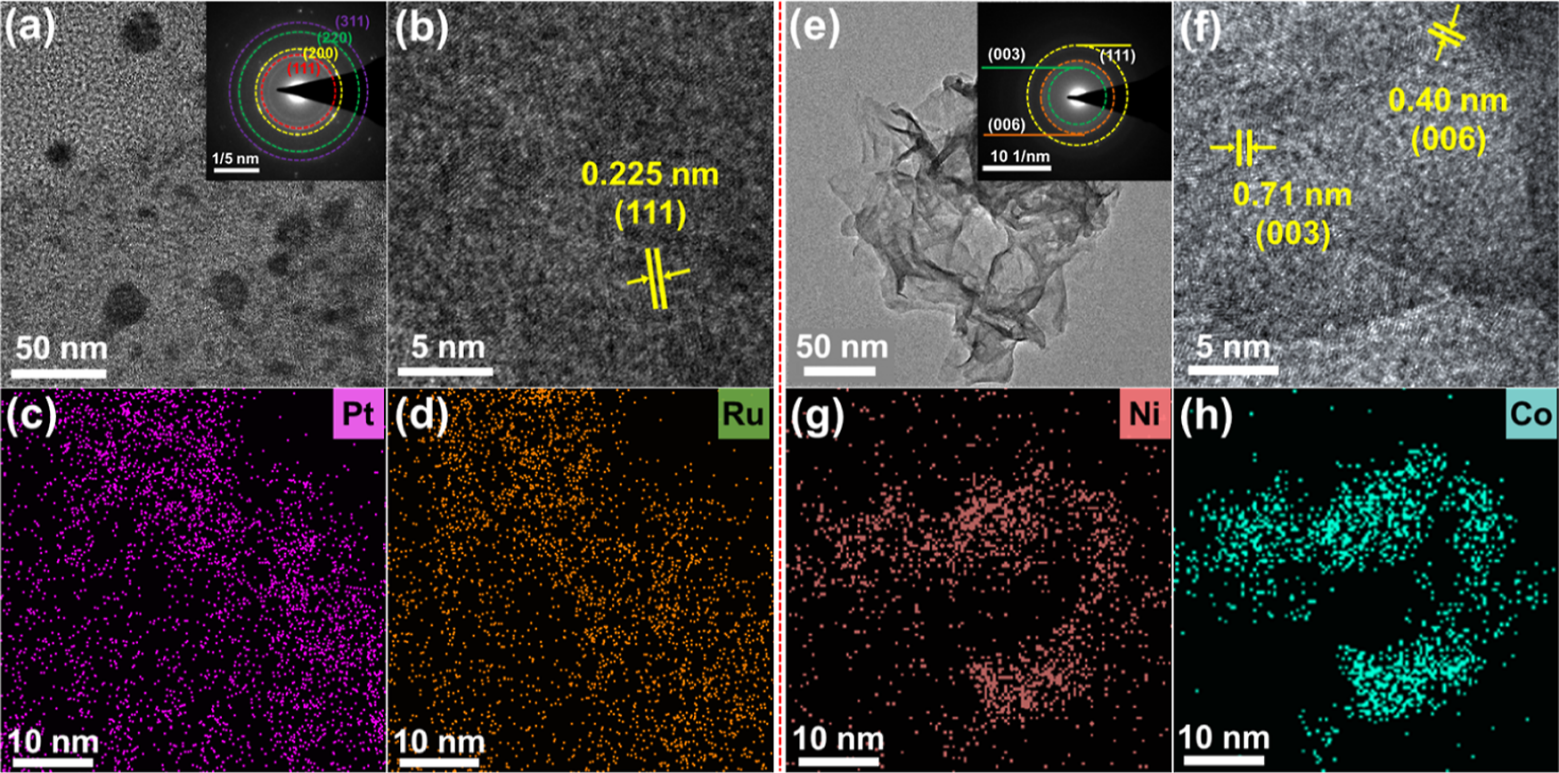
Electron microscopy images of the PtRu alloy and NiCo(OOH)_*x*_ nanosheets. (a) TEM image with SAED pattern of the PtRu alloy. (b) HRTEM image. EDS mapping of (c) Pt and (d) Ru in the PtRu alloy. (e) TEM image with SAED pattern of NiCo(OOH)_*x*_ nanosheets. (f) HRTEM image. EDS mapping of (g) Ni and (h) Co in NiCo(OOH)_*x*_ nanosheets.

To clarify the surface electronic characteristics of the PtRu alloy, transmission electron microscopy (TEM) was used to measure the morphology and structural characteristics of the sample. A representative TEM image of the PtRu alloy is depicted in Figure 2a. The particles within the highly alloyed sample were found to be on the nanometer scale. High-resolution TEM (HRTEM) images, as shown in Figure 2b, revealed distinct lattice fringes with a spacing of 0.225 nm, which was indicative of the typical (111) plane. Selected area electron diffraction (SAED) patterns (Figure 2a) displayed obvious diffraction rings that were in complete congruence with the characteristic peaks observed in the XRD results. Energy dispersive X-ray spectroscopy (EDS) mapping images for individual nanoparticles are illustrated in Figure 2c,d, which demonstrate uniform distributions of Pt and Ru throughout the particles, with a notable overlap between the two elements. This homogeneous distribution provided further evidence of the alloying feature of the PtRu alloy. The nanosheets on the staggered wires in the NiCo(OOH)_*x*_ sample are shown in Figure S2. The atomic-level crystal structure of the NiCo(OOH)_*x*_ nanosheets was further investigated using TEM. HRTEM images and SAED patterns revealed correspondence to the (003), (006), and (111) planes of the hydroxide phase (Figure 2e,f). EDS mapping demonstrated that Ni and Co elements were evenly distributed throughout the NiCo(OOH)_*x*_ nanosheets (Figure 2g,h).

### Electrocatalytic Reduction of HMF

First, electrochemical tests were conducted using a PtRu alloy as the cathode without adding any reaction substrates to evaluate its electrochemical performance in different electrolytes. Linear sweep voltammetry (LSV) curves and *C*_dl_ values were obtained to assess the performance of the PtRu alloy in various electrolytes, as shown in Figures S3 and S4. The potentiostatic tests also indicated that the PtRu alloy maintains long-term durability at various potentials, as shown in Figure S5. Based on the HER test results, it was evident that the PtRu alloy exhibited significant HER performance in both acidic and alkaline electrolytes. However, under mildly neutral conditions, the HER performance was somewhat suppressed, which suggested that neutral conditions might favor the occurrence of HMF reduction reactions.

**Figure 3:**
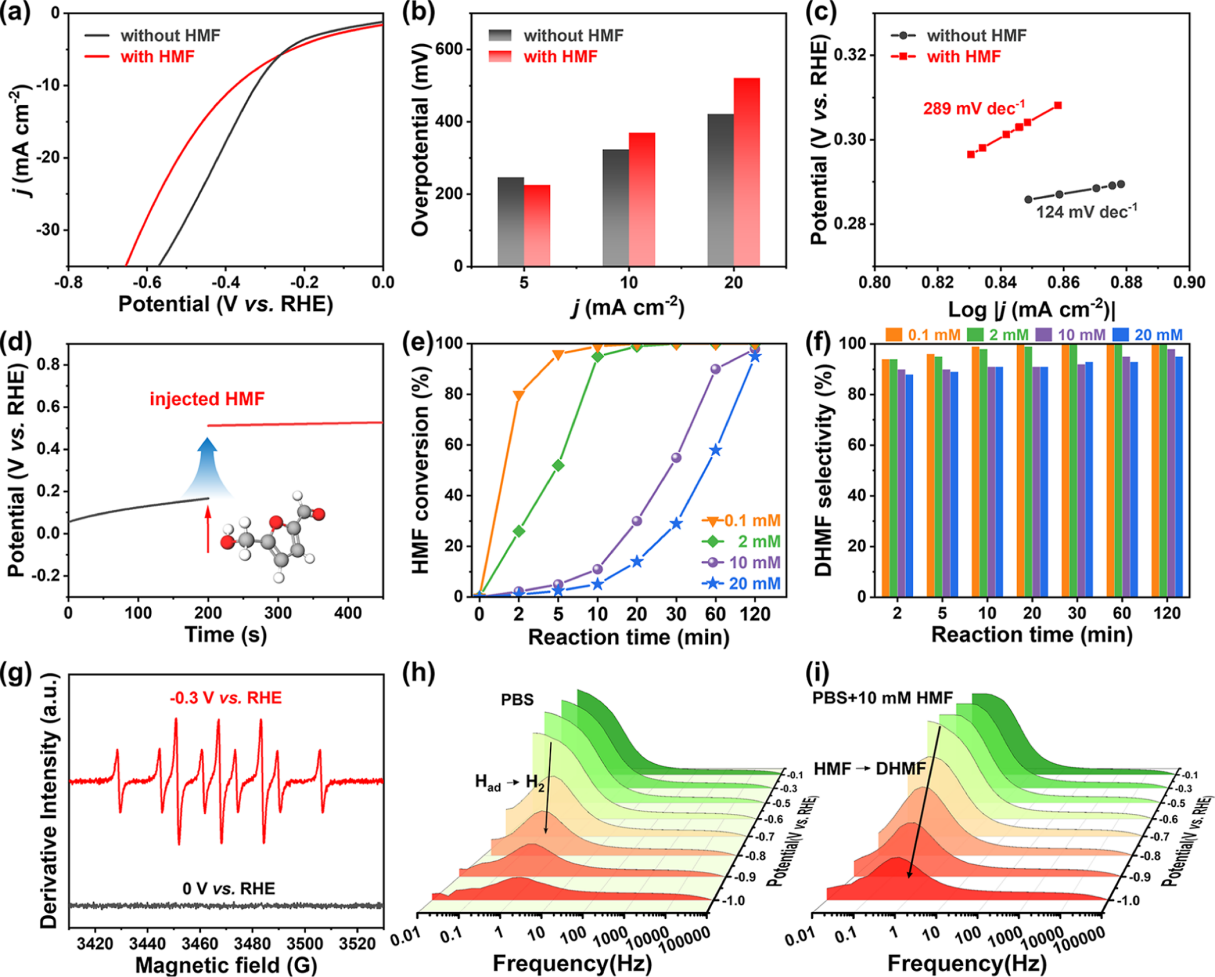
Electrocatalytic HMF reduction performance. (a) LSV curves of the PtRu alloy in 0.1 M PBS (pH of 7.4) with HMF at various ranges of potential with a scan rate of 50 mV s^–1^. (b) Overpotential and (c) Tafel slope of the PtRu alloy. (d) The OCP of the PtRu alloy in 0.1 M PBS solution before and after HMF was injected. (e) HMF conversion. (f) DHMF selectivity. (g) DMPO spin-trapping EPR spectra of electrocatalytic reduction with and without HMF. Bode plots (h) without and (i) with 10 mM HMF in PBS solution at different potentials (−0.2 ∼ −1.2 V vs RHE).

Next, the electrocatalytic reduction of HMF was investigated. Under neutral conditions (0.1 M PBS containing 1.37 M NaCl, 0.0512 M KCl, 0.101 M Na_2_HPO_4_, and 0.0176 M KH_2_PO_4_), the electrocatalytic reduction of HMF was studied in the presence and absence of HMF. As shown in Figure 3a, after adding HMF, the current density in blank PBS electrolyte was significantly inhibited, indicating that the electrocatalytic reduction of HMF could restrain the HER. The overpotential and Tafel slope (Figure 3b,c) increased after the addition of HMF, which can be attributed to the adsorption of HMF on the surface of the PtRu alloy instead of H^+^/H_3_O^+^. Moreover, the open-circuit potential (OCP) was measured to monitor changes in the organic adsorbent content within the inner Helmholtz layer.^34,35^ Upon injection of HMF, a significant OCP change of 0.42 V was observed in the PtRu alloy, suggesting increased adsorption of HMF molecules (Figure 3d). The reaction was then studied to understand the electrocatalytic reduction process in an H-type electrocatalytic cell at a constant potential. As shown in Figure 3e, HMF conversion was investigated with various concentrations of HMF, including 0.1, 2, 10, and 20 mM, at a standard temperature (25 °C). HMF was completely electrocatalytically hydrogenated to obtain DHMF in 10 min with 0.1 mM HMF, and the DHMF selectivity was ∼100% (Figure 3f). When 10 mM HMF was added, the HMF conversion exceeded 90% after 60 min, superior the aldehydes hydrogenation performance using traditional method.^36,37^ Furthermore, the FE of the reaction system consistently remained above 95% (Figure S6).

To better understand the changes in H* and HMF adsorption, in situ EPR spin trapping was applied to investigate the participation of H* in electrocatalytic reduction (Figure 3g). The generation of H* was indicated by the nine distinct peaks of DMPO-H observed in PBS containing HMF at a current density of 10 mA cm^–2^. Conversely, at a current density of 0 mA cm^–2^, the signals were absent. To gain a deeper understanding of the electrocatalytic reduction characteristics of the PtRu alloy electrode, EIS was performed. As shown in Figure 3h, the Bode plots for the HER on the PtRu alloy electrode exhibited prominent peaks at intermediate and low frequencies. The peak observed at low frequencies (<1 Hz) corresponded to the Volmer step, indicating that the adsorption of HMF is a crucial step.^38^ The other peak was found in the 1–10 Hz range and was connected to the Heyrovsky step, implying that desorption of the DHMF product is the rate-determining step. As the applied potentials rose, the peak of the Volmer step was overwhelmed by the four-part equivalent circuit. Then, with the addition of HMF, the phase degree (Figure 3i) of the medium-to-low frequency (1–100 Hz) over the PtRu alloy electrode increased, implying the effect of HMF and active species adsorption.

### Pairing of Electrocatalytic Reduction and Oxidation of HMF

**Figure 4:**
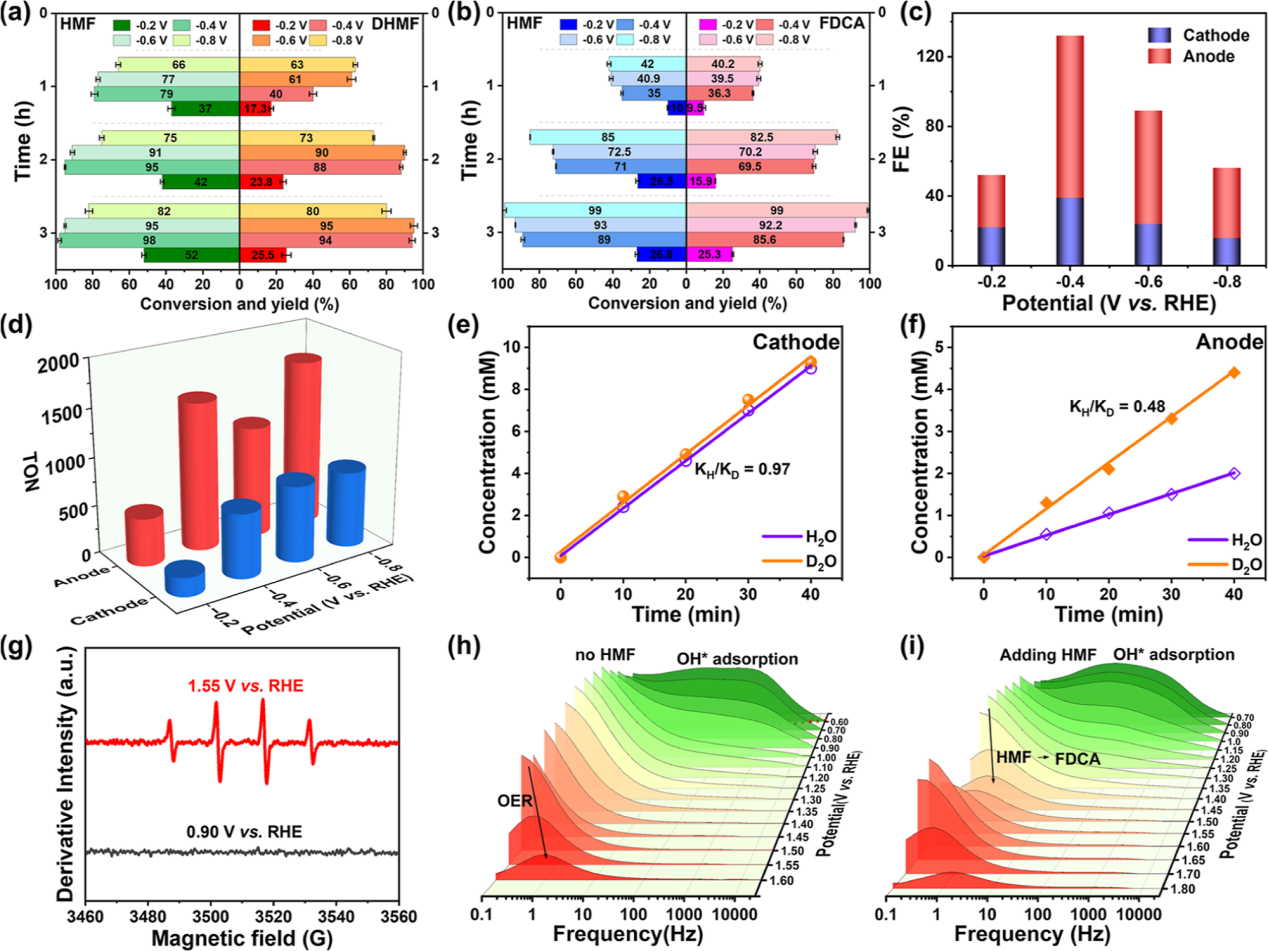
Pairing system for HMF reduction and oxidation: The cathode electrolyte was 0.1 M PBS, and the anode electrolyte was 1 M KOH. (a) Conversion rate of the cathode HMF and yield of DHMF products at −0.2, −0.4, −0.6, and −0.8 V vs RHE. (b) HMF conversion and FDCA yield at −0.2, −0.4, −0.6, and −0.8 V vs RHE. (c) FE and (d) TON at −0.2, −0.4, −0.6, −0.8 V vs RHE. The kinetic isotope effects of (e) DHMF at the cathode and (f) FDCA at the anode during the PERO process. Test conditions: −0.8 V vs RHE, 0.1 M PBS, and 10 mM HMF. (g) EPR analysis of NiCo(OOH)_*x*_ nanosheets at the anode at 1.55 and 0.90 V vs RHE during the HMF electrooxidation reaction. The reaction mixture was tested by simultaneous EPR. Bode plots of NiCo(OOH)_*x*_ nanosheets in KOH (h) without and (i) with HMF at different potentials obtained via operando EIS analysis.

Pairing electrocatalysis involves pairing two advantageous half-reactions to enhance energy efficiency and produce valuable products.^32,39,40^ To better understand the change in the pairing reaction, the transformation effects under different application potentials were explored. The HMF conversion and DHMF yield on the PtRu alloy at four different potentials of −0.2, −0.4, −0.6, and −0.8 V vs RHE using the cathode control terminal are shown in Figure 4a. When a low potential (−0.2 V vs RHE) was applied, the current density was only 5 mA cm^–2^, corresponding to the initial stage of the linear cycle curve. The PtRu alloy electrode obtained a low HMF conversion and DHMF yield of less than 30%. When the applied potential was increased to −0.4 V vs RHE, the conversion of HMF was more than two times greater than that at −0.2 V vs RHE, and the yield was as high as 94%. When the applied potential was increased to −0.8 V vs RHE, the HMF conversion and DHMF yield did not increase further. In Figure 4b, the difference between the HMF conversion and FDCA yield for electrocatalytic oxidation at the anode is displayed. The anode NiCo(OOH)_*x*_ nanosheets showed low HMF conversion and FDCA yield at low potential (initial potential), indicating that the current was insufficient to produce efficient oxidation of HMF. When the potential increased to −0.4 V vs RHE, the electrocatalytic oxidation reactivity was remarkable, and the conversion of HMF suddenly increased 3–4 times to 89%, which was much greater than the 26% observed at −0.2 V vs RHE, at which point the HMF was completely converted to FDCA. When the potential reached −0.8 V vs RHE, nearly full conversion of HMF and perfect selectivity of FDCA were achieved. Compared with the electrocatalytic conversion of HMF in the three-electrode system, the time required for complete oxidation of HMF in the pairing reaction increased, but the reduction rate of HMF in the cathode accelerated, and 95% of the DHMF product was obtained in 1 h. These results showed that DHMF produced at the cathode was faster than that at the anode in a short time when the substrate concentration was sufficient. The electrocatalytic oxidation at the anode was carried out step by step, and nearly 100% conversion was obtained after 3 h of reaction. The hydrogenation product DHMF and oxidation product FDCA were obtained by pairing the electrochemical oxidation and reduction of HMF. After 5 h of pairing, the FE density of the cathode and anode was greater than 130% at −0.4 V vs RHE (Figure 4c). The potential dependent turnover number (TON) of the pairing reaction was further calculated to evaluate the pairing efficiency more accurately (Figure 4d). The TON value of the cathode was 790, and TON value of the anodic oxidation end was 1721. The turnover frequency (TOF) was subsequently used to measure the catalytic reaction rate, where the TOF of the cathode was 248 h^–1^ and that of the anode was 595 h^–1^.

Regarding the oxidative half-reaction, a new reaction pathway was identified. As shown in Figure S7, 2,5-diformylfuran (DFF) was observed during PERO at 0.08 h; however, its amount gradually decreased during subsequent electrolysis. This indicates that DFF, containing an aldehyde group, is highly unstable in alkaline electrolytes and rapidly undergoes the Cannizzaro reaction, resulting in the formation of carboxylic acid and alcohol molecules.^41^ When DFF was added to the electrolyte, HMF and FFCA were detected over time, further confirming that nonelectrochemical reactions involving DFF occurred. In contrast, the HMFCA intermediate remained unchanged under alkaline conditions. Therefore, the oxidation path of HMF to FDCA primarily involves the formation of HMFCA intermediates, but also includes the nonelectrochemical oxidation process of the DFF intermediate (Figure S8).

To verify the changes in the rate-controlled steps after the addition of HMF, the KIEs of the electrocatalytic reduction/oxidation reactions were measured using H/D isotope substitution tests. KIE refers to the change in reaction rate when an atom in a reactant was replaced by its isotope. This occurs if the alteration in reactants does not counterbalance the change in the transition state. Specifically, according to transition state theory, the reaction rate will vary if there is a difference in the ground state vibration energy between the reactants and the transition state before and after isotope substitution.^42^ In the isotope substitution experiment, buffer salts were dissolved in H_2_O and D_2_O, and a pairing reaction was performed after HMF was added. A KIE of ∼1 for electrocatalytic reduction was observed at the cathode in Figure 4e, following zero-order dynamics, indicating that the isotope-substituted bond (O–H) did not change during the determination step. The rate-controlled step was a proton-independent electron transfer process, and a shift in the substrate concentration had little effect on the reaction rate. It was assumed that surface adsorption on the PtRu alloy electrode was a rate-controlled step during the pairing reaction, which is consistent with the Tafel slopes.^43^ In contrast, for the KIE value (≪1) for electrocatalytic oxidation in Figure 4f, the oxidation reaction was actually faster with isotope substitution than without isotope substitution. As the vibration became slower, the force constant of the bending vibration became larger during the transition state. In this case, the difference in the zero-point energy at the transition state was greater than that at the reactant state, which was an opposite kinetic isotope effect.^41^ The isotope substitution bond (O–H) of the rate-controlled step was not broken when the transition state changed, which was the second-order kinetic isotope effect. This was attributed to hybridization or participation in conjugation (i.e., carbon ions adjacent to the O–H/O–D bonds). The magnitude of the isotopic effect was much smaller than that of the primary KIE, and the transition state was generated late. The inherent inverse reduction eliminated the isotopic effect at the transition state. Therefore, it was speculated that the rate-determining step involves the O–H/O–D bonds participating in conjugation. The inverse KIE value was also consistent with the multistep oxidation-addition mechanism of HMF at the anode. To further explore the multistep oxidation–addition process of electrocatalytic oxidation, the active species of OH* were investigated using EPR. As shown in Figure 4g, the PERO of HMF showed a strong DMPO-OH signal at 1.55 V vs RHE at the anode. This suggested that the formation of OH* active sites was the main mechanism for the electrocatalytic oxidation of HMF on NiCo(OOH)_*x*_ nanosheets. In addition, the degree of OH* adsorption on the anode electrode was studied using EIS, and Bode plots are presented in Figure 4h,i. On the surface of the NiCo(OOH)_*x*_ electrode, the formation of adsorbed OH* intermediates occurred, which then interacted with HMF, a nucleophile, resulting in the production of FDCA.^44^

### Universality of the Pairing System

**Figure 5:**
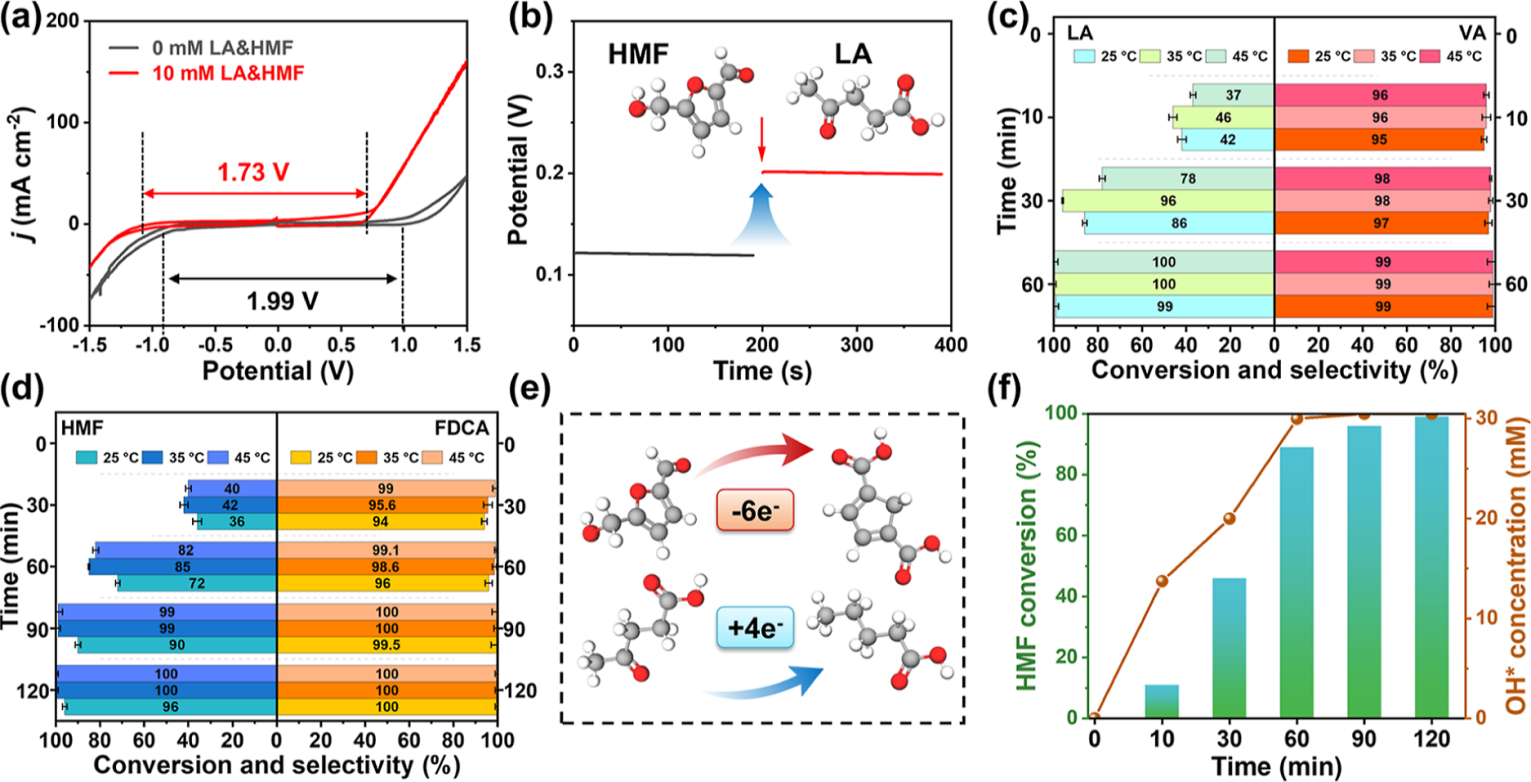
Performance for multiple coupled reaction systems. (a) Cyclic voltammetry (CV) curves at potential ranges of −1.5–1.5 V in PBS with 10 mM LA (cathode) and 10 mM HMF (anode). (b) OCP in an H-type cell. (c) LA conversion and VA selectivity for electrocatalytic reduction in PBS with 10 mM LA at the cathode. (d) HMF conversion and FDCA selectivity in KOH with 10 mM HMF at the anode. (e) Schematic diagram of the oxidation of HMF to FDCA and the reduction of LA to VA. (f) Changes in HMF conversion and OH concentration with reaction time.

To study the universality of the proposed method, the pairing reaction of biomass-derived levulinic acid (LA) reduction with HMF oxidation was investigated in H-type cells. In the system, a cell potential of 1.99 V was required for the HER–OER to reach a current density of 10 mA cm^–2^. Notably, by adding 10 mM LA at the cathode and 10 mM HMF at the anode, the system required only 1.73 V to reach the same current density, as shown in Figure 5a. In addition, the OCP increased by 0.09 V after adding LA and HMF, indicating a substantial change in the electrode potential equilibrium (Figure 5b). This variation might result from the interchange of adsorbates and ions in the Helmholtz layer, indicating significant adsorption of LA and HMF on the cathode and anode surfaces. Potentiostatic tests of the PtRu alloy were further performed at 25, 35, and 45 °C (Figure S9). For the electrocatalytic reduction at the cathode (Figure 5c), more than 99% LA conversion and 99% valeric acid (VA) selectivity were obtained after 60 min for pairing reactions at −1.4 V at 25 °C. At 35 and 45 °C, the LA conversion and VA selectivity reached ∼100%. At the anode, the oxidation rate of HMF showed a small effect as the temperature of the system increased, as shown in Figure 5d. After 90 min of reaction, the NiCo(OOH)_*x*_ electrode showed an HMF conversion rate of more than 90% and an FDCA selectivity of nearly 99%. According to the experimental data, the VA product from LA reduction was followed by four electrons, while the most positive extreme HMF oxidation was FDCA followed by six electrons (Figure 5e). To clarify the concentration of active substances, the concentration of hydroxyl radicals at the anode was determined. Among them, OH* was responsible for the electrocatalytic oxidation of HMF.^32,45^ Therefore, we further investigated the generation of OH* in the pairing system using benzyl alcohol as a probe molecule.^46^ As shown in Figure 5f, when the concentration of OH* increased to 30 mM within 60 min, 9.5 mM OH* was reached, which was close to the theoretical value. These data further supported the fact that OH* was the active site in the electrocatalytic oxidation of HMF.

The pairing reactions with LA reduction || furfural (FF) oxidation and LA reduction || benzaldehyde (BAE) oxidation were then investigated in the H-type cell. As shown in Figure S10a, the oxidation curve shifted toward the *Y*-axis after adding 10 mM FF at the anode, but the curve did not change after adding 20 mM LA at the cathode. After 60 min of the pairing reaction at −1.4 V, the PtRu alloy electrode showed more than 99% LA conversion and VA selectivity at the cathode (Figure S10b), and the NiCo(OOH)_*x*_ nanosheets electrode showed 90% conversion of FF and 98% furoic acid (FurAc) selectivity at the anode, as shown in Figure S10c. According to the experimental data, we found a small amount of the product gamma-valerolactone (GVL) with a selectivity of <5%, as shown in Figure S10d, indicating that the main product for LA reduction was VA at the cathode. Moreover, the pairing reaction of LA reduction || BAE oxidation resulted in the full conversion of LA (Figure S11a) and benzoic acid (BAD) (Figure S11b) after 60 min. Furthermore, an FE of ∼95% was attained for each side. Table S1 lists relevant studies on pairing electrocatalytic reduction and oxidation of biomass-derived platform chemicals into highly value-added chemicals. With the aid of a PtRu alloy and NiCo(OOH)_*x*_ nanosheets, the multiple reaction systems investigated in this study exhibited highly efficient performance.

**Figure 6:**
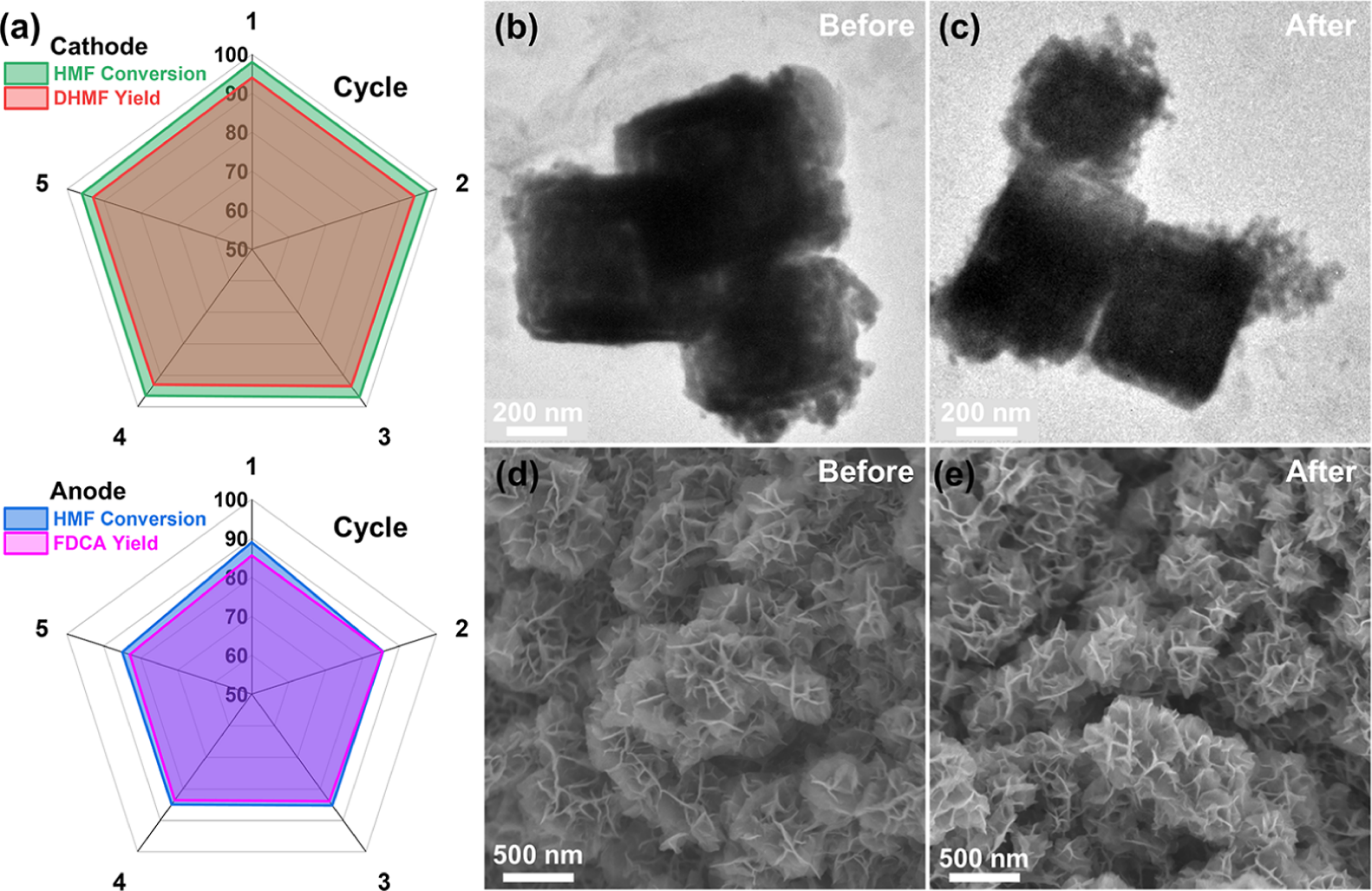
Durability of cathode and anode. (a) The catalytic performance of five cycles of PERO using the recycled PtRu alloy at the cathode and NiCo(OOH)_*x*_ nanosheets at the anode. SEM images of the PtRu alloy (b) before and (c) after PERO. SEM images of NiCo(OOH)_*x*_ nanosheets (d) before and (e) after PERO.

Furthermore, the catalytic performance of PERO was evaluated using a recycled PtRu alloy electrode at the cathode and a NiCo(OOH)_*x*_ electrode at the anode (Figure 6a). After more than five runs of PERO, pairing an electrolyzer with electrocatalytic reduction and oxidation of HMF resulted in more than 80% HMF conversion, 93% DHMF yield and 85% FDCA yield. When the reaction system was expanded to include the reduction of LA paired with the oxidation of HMF, the conversion rates of both LA and HMF remained close to 100%, even after five cycles, with a product selectivity higher than 95% (Figure S12). The CV curves (Figure S13) showed no changes after five cycles in the LA reduction || HMF oxidation system. Compared to those of the fresh electrode, the SEM images of the PtRu alloy (Figure 6b,c) and NiCo(OOH)_*x*_ nanosheets (Figure 6d,e) after using PERO showed no significant morphological changes. Based on the results from the tests of the paired electrocatalytic reaction system, it was evident that rationally pairing the cathode and anode to achieve effective electrocatalytic conversion holds significant potential for the simultaneous production of high-value chemicals.

## Conclusions

In summary, we successfully constructed a pairing reaction system for high-efficiency electrocatalytic HMF reduction and oxidation. The PtRu alloy electrocatalyst was first synthesized via a solvent-free microwave-assisted method for high-performance electrocatalytic reduction of biomass-derived HMF. After the addition of HMF, the bipolarity conversion rate of HMF was nearly perfect, the conversion rate of HMF was 98%, and the DHMF yield was 94% at the cathode. The positive extreme HMF was almost completely transformed, and the FDCA yield was 86% using NiCo(OOH)_*x*_ nanosheets at the anode. A coupled electrocatalytic oxidation and reduction system can obtain more than 130% FE, the TON value of the cathode is 790, and the TON value of the anode is 1721, which is far superior to most of the previously reported candidates. The isotope substitution tests revealed that the cathodic reduction reaction was a proton-independent electron transfer process, while the anodic reaction was a proton-involved multipole reaction, which also proved that the electrocatalytic oxidation of HMF was a multistep oxidation mechanism. We clarified the critical role of H* on the PtRu alloy for reduction and the OH* active sites on the NiCo(OOH)_*x*_ nanosheets electrode for oxidation. Therefore, coupling the oxidation and reduction reactions of HMF under cathodic potential control is a feasible solution for upgrading high-value chemicals. The PERO system was also universal and ideal for pairing LA reduction || HMF oxidation, LA reduction || FF oxidation, and LA reduction || BAE oxidation. The paired system developed in this work could be beneficial for various biomass refineries.

## Methods

### Controllable Synthesis of the PtRu Alloy

In a typical synthesis process, equal masses of platinum(II) acetylacetonate (Pt(acac)_2_) and ruthenium(III) acetylacetonate (Ru(acac)_3_), each weighing 14 mg, were meticulously ground for 30 min to form a homogenized precursor mixture with a uniform color and texture. The admixture was then exposed to microwave-assisted heating under an atmosphere of H_2_/Ar. Initially, the mixture was heated at a microwave power of 260 W, ramping the temperature to 200 °C at an average heating rate of 10 °C min^–1^, and maintained at this temperature for 1 h. In the subsequent heat treatment step, the temperature was rapidly increased to 400 °C at an average heating rate of 100 °C min^–1^, using a microwave power of 1300 W, and held for 20 min. After the microwave irradiation was completed, the sample cooled to room temperature over 9 h, yielding a homogeneously distributed PtRu alloy.

### Controllable Synthesis of the NiCo(OOH)_*x*_

First, 0.5 M Co^2+^ and 0.5 M Ni^2+^ solutions were prepared, along with 1 M NaOH, and 0.1 M Na_2_CO_3_ solutions. Subsequently, 5 mL of 0.5 M Ni^2+^ and 10 mL of 0.5 M Co^2+^ were mixed in 35 mL of ultrapure water with ultrasound (denoted as solution A). A buffer solution B containing 1 M NaOH and 0.1 M Na_2_CO_3_ in a 4:1 ratio was prepared. The pH of solution A was adjusted to 7.5 using solution B. Afterward, a pretreated Ni foam electrode (1 cm^2^) was placed in 35 mL of the mixed solution. The electrodeposition was performed using a galvanostatic deposition method on an Autolab 204 workstation. Finally, the Ni foam with the deposited catalysts was dried in a vacuum oven at 60 °C for 5 h.

### Pairing Reactions

For reduction reaction, the electrolytes used included 1 M pKOH solution (pH 13.6), 0.5 M H_2_SO_4_ (pH 0.38), and 0.01 M PBS (pH 7.4). Then, 30 mL of electrolyte was added to both sides of an H-type cell (50 mL total volume) with a Nafion 117 membrane. The working electrode was a PtRu alloy on carbon fiber paper, while a Pt wire (Φ = 1 mm, 4 cm in length, Shanghai CH Instruments Ins.) was used as the counter electrode in all electrochemical experiments. The LSV curves were obtained in the potential range of 0.2 to −0.9 V_RHE_ at a scan rate of 10 mV s^–1^.

For pairing reactions, electrochemical testing was conducted using an Autolab M204 (Metrohm) instrument within the cathode and anode electrode configuration shown in Scheme S1. In these tests, the cathode electrode was PtRu alloy on carbon fiber paper, placed in 0.1 M PBS electrolyte, while the anode electrode was NiCo(OOH)_*x*_ nanosheets in 1 M KOH electrolyte. The CV was conducted at a scan rate of 10 mV s^–1^ within a potential range of −1.5–1.5 V. The combination of electrochemical reduction and oxidation reactions was carried out in a 10 mM HMF solution. The coupled electrochemical reduction and oxidation reactions were performed at a constant potential/current. All experiments were carried out at 25 °C and 101.95 kPa. Further details of the experiments can be found in the Supporting Information.

## Supporting Information Available

Experimental details including chemicals, characterization techniques, electrochemical measurements, product analysis, and additional graphs of experimental results (PDF)

## References

[ref1] Luterbacher J. S.; Rand J. M.; Alonso D. M.; Han J.; Youngquist J. T.; Maravelias C. T.; Pfleger B. F.; Dumesic J. A. Nonenzymatic sugar production from biomass using biomass-derived γ-valerolactone. Science 2014, 343 (6168), 277–280. 10.1126/science.1246748. 24436415

[ref2] Liu S.; Jin Y.; Huang S.; Zhu Q.; Shao S.; Lam J. C.-H. One-pot redox cascade paired electrosynthesis of gamma-butyrolactone from furoic acid. Nat. Commun. 2024, 15 (1), 1141 10.1038/s41467-024-45278-z. 38326323 PMC10850494

[ref3] Liu C.; Wang H.; Karim A. M.; Sun J.; Wang Y. Catalytic fast pyrolysis of lignocellulosic biomass. Chem. Soc. Rev. 2014, 43 (22), 7594–7623. 10.1039/C3CS60414D. 24801125

[ref4] You B.; Sun Y. Innovative strategies for electrocatalytic water splitting. Acc. Chem. Res. 2018, 51 (7), 1571–1580. 10.1021/acs.accounts.8b00002. 29537825

[ref5] Wang T.; Tao L.; Zhu X.; Chen C.; Chen W.; Du S.; Zhou Y.; Zhou B.; Wang D.; Xie C.; et al. Combined anodic and cathodic hydrogen production from aldehyde oxidation and hydrogen evolution reaction. Nat. Catal. 2022, 5 (1), 66–73. 10.1038/s41929-021-00721-y.

[ref6] Liu S.; Zhang B.; Yang Z.; Xue Z.; Mu T. Deep eutectic solvothermal NiS_2_/CdS synthesis for the visible-light-driven valorization of the biomass intermediate 5-hydroxymethylfurfural (HMF) integrated with H_2_ production. Green Chem. 2023, 25 (7), 2620–2628. 10.1039/D2GC04535D.

[ref7] Kloth R.; Vasilyev D. V.; Mayrhofer K. J. J.; Katsounaros I. Electroreductive 5-hydroxymethylfurfural dimerization on carbon electrodes. ChemSusChem 2021, 14 (23), 5245–5253. 10.1002/cssc.202101575. 34549892 PMC9298403

[ref8] Jain A. B.; Vaidya P. D. Kinetics of catalytic hydrogenation of 5-hydroxymethylfurfural to 2,5-bis-hydroxymethylfuran in aqueous solution over Ru/C. Int. J. Chem. Kinet. 2016, 48 (6), 318–328. 10.1002/kin.20992.

[ref9] Frontana-Uribe B. A.; Little R. D.; Ibanez J. G.; Palma A.; Vasquez-Medrano R. Organic electrosynthesis: a promising green methodology in organic chemistry. Green Chem. 2010, 12 (12), 2099–2119. 10.1039/c0gc00382d.

[ref10] Sperry J. B.; Wright D. L. The application of cathodic reductions and anodic oxidations in the synthesis of complex molecules. Chem. Soc. Rev. 2006, 35 (7), 605–621. 10.1039/b512308a. 16791332

[ref11] Yoshida J.-I.; Kataoka K.; Horcajada R.; Nagaki A. Modern strategies in electroorganic synthesis. Chem. Rev. 2008, 108 (7), 2265–2299. 10.1021/cr0680843. 18564879

[ref12] Roylance J. J.; Kim T. W.; Choi K.-S. Efficient and selective electrochemical and photoelectrochemical reduction of 5-hydroxymethylfurfural to 2,5-bis(hydroxymethyl)furan using water as the hydrogen source. ACS Catal. 2016, 6 (3), 1840–1847. 10.1021/acscatal.5b02586.

[ref13] Zhang W.; Qi Y.; Zhao Y.; Ge W.; Dong L.; Shen J.; Jiang H.; Li C. Rh-dispersed Cu nanowire catalyst for boosting electrocatalytic hydrogenation of 5-hydroxymethylfurfural. Sci. Bull. 2023, 68 (19), 2190–2199. 10.1016/j.scib.2023.07.036. 37580202

[ref14] Sanghez de Luna G.; Ho P. H.; Lolli A.; Ospitali F.; Albonetti S.; Fornasari G.; Benito P. Ag electrodeposited on Cu open-cell foams for the selective electroreduction of 5-hydroxymethylfurfural. ChemElectroChem 2020, 7 (5), 1238–1247. 10.1002/celc.201902161.

[ref15] Zhang Y.; Wang B.; Qin L.; Li Q.; Fan Y. A non-noble bimetallic alloy in the highly selective electrochemical synthesis of the biofuel 2,5-dimethylfuran from 5-hydroxymethylfurfural. Green Chem. 2019, 21 (5), 1108–1113. 10.1039/C8GC03689F.

[ref16] Tan J.; Zhang W.; Shu Y.; Lu H.; Tang Y.; Gao Q. Interlayer engineering of molybdenum disulfide toward efficient electrocatalytic hydrogenation. Sci. Bull. 2021, 66 (10), 1003–1012. 10.1016/j.scib.2020.11.002. 36654245

[ref17] Ji K.; Xu M.; Xu S.-M.; Wang Y.; Ge R.; Hu X.; Sun X.; Duan H. Electrocatalytic hydrogenation of 5-hydroxymethylfurfural promoted by a Ru_1_Cu single-atom alloy catalyst. Angew. Chem., Int. Ed. 2022, 61 (37), e202209849 10.1002/anie.202209849. 35876073

[ref18] Zhang Y.; Shen Y. Electrochemical hydrogenation of levulinic acid, furfural and 5-hydroxymethylfurfural. Appl. Catal., B 2024, 343, 123576 10.1016/j.apcatb.2023.123576.

[ref19] Yang Y.; Yu Y.; Li J.; Chen Q.; Du Y.; Rao P.; Li R.; Jia C.; Kang Z.; Deng P.; et al. Engineering ruthenium-based electrocatalysts for effective hydrogen evolution reaction. Nano-Micro Lett. 2021, 13 (1), 160 10.1007/s40820-021-00679-3. PMC8310550 34302536

[ref20] Wang H.; Chen S.; Wang C.; Zhang K.; Liu D.; Haleem Y. A.; Zheng X.; Ge B.; Song L. Role of Ru oxidation degree for catalytic activity in bimetallic Pt/Ru nanoparticles. J. Phys. Chem. C 2016, 120 (12), 6569–6576. 10.1021/acs.jpcc.5b12267.

[ref21] Kormányos A.; Speck F. D.; Mayrhofer K. J. J.; Cherevko S. Influence of fuels and pH on the dissolution stability of bifunctional PtRu/C alloy electrocatalysts. ACS Catal. 2020, 10 (19), 10858–10870. 10.1021/acscatal.0c02094.

[ref22] Russell A. E.; Rose A. X-ray absorption spectroscopy of low temperature fuel cell catalysts. Chem. Rev. 2004, 104 (10), 4613–4636. 10.1021/cr020708r. 15669164

[ref23] Hulicova-Jurcakova D.; Seredych M.; Lu G. Q.; Bandosz T. J. Combined effect of nitrogen- and oxygen-containing functional groups of microporous activated carbon on its electrochemical performance in supercapacitors. Adv. Funct. Mater. 2009, 19 (3), 438–447. 10.1002/adfm.200801236.

[ref24] Li T.; Cao Y.; He J.; Berlinguette C. P. Electrolytic CO_2_ reduction in tandem with oxidative organic chemistry. ACS Cent. Sci. 2017, 3 (7), 778–783. 10.1021/acscentsci.7b00207. 28776020 PMC5532713

[ref25] Zhang P.; Sheng X.; Chen X.; Fang Z.; Jiang J.; Wang M.; Li F.; Fan L.; Ren Y.; Zhang B.; et al. Paired electrocatalytic oxygenation and hydrogenation of organic substrates with water as the oxygen and hydrogen source. Angew. Chem., Int. Ed. 2019, 58 (27), 9155–9159. 10.1002/anie.201903936. PMC6617801 31025774

[ref26] Wang Y.; Zhang M.; Liu Y.; Zheng Z.; Liu B.; Chen M.; Guan G.; Yan K. Recent advances on transition-metal-based layered double hydroxides nanosheets for electrocatalytic energy conversion. Adv. Sci. 2023, 10 (13), 2207519 10.1002/advs.202207519. PMC10161082 36866927

[ref27] Wang C.; Wu Y.; Bodach A.; Krebs M. L.; Schuhmann W.; Schüth F. A novel electrode for value-generating anode reactions in water electrolyzers at industrial current densities. Angew. Chem., Int. Ed. 2023, 62 (7), e202215804 10.1002/anie.202215804. PMC10107951 36440966

[ref28] Zhang M.; Liu Y.; Liu B.; Chen Z.; Xu H.; Yan K. Trimetallic NiCoFe-layered double hydroxides nanosheets efficient for oxygen evolution and highly selective oxidation of biomass-derived 5-hydroxymethylfurfural. ACS Catal. 2020, 10 (9), 5179–5189. 10.1021/acscatal.0c00007.

[ref29] Qi J.; Du Y.; Yang Q.; Jiang N.; Li J.; Ma Y.; Ma Y.; Zhao X.; Qiu J. Energy-saving and product-oriented hydrogen peroxide electrosynthesis enabled by electrochemistry pairing and product engineering. Nat. Commun. 2023, 14 (1), 6263 10.1038/s41467-023-41997-x. 37805528 PMC10560254

[ref30] Rodríguez-Gómez A.; Lepre E.; Sánchez-Silva L.; López-Salas N.; de la Osa A. R. PtRu nanoparticles supported on noble carbons for ethanol electrooxidation. J. Energy Chem. 2022, 66, 168–180. 10.1016/j.jechem.2021.07.004.

[ref31] Jiang J.; Zhang A.; Li L.; Ai L. Nickel–cobalt layered double hydroxide nanosheets as high-performance electrocatalyst for oxygen evolution reaction. J. Power Sources 2015, 278, 445–451. 10.1016/j.jpowsour.2014.12.085.

[ref32] Zhang M.; Xu Z.; Liu B.; Duan Y.; Zheng Z.; Li L.; Zhou Q.; Matveeva V. G.; Hu Z.; Yu J.; et al. Anchoring hydroxyl intermediate on NiCo(OOH)_*x*_ nanosheets to enable highly efficient electrooxidation of benzyl alcohols. AIChE J. 2023, 69 (7), e18077 10.1002/aic.18077.

[ref33] Gao M.; Huang J.; Liu Y.; Li X.; Wei P.; Yang J.; Shen S.; Cai K. Electrochemically finely regulated NiCo-LDH/NiCoOOH nanostructured films for supercapacitors with record high mass loading, areal capacity, and energy density. Adv. Funct. Mater. 2023, 33 (51), 2305175 10.1002/adfm.202305175.

[ref34] Heidary N.; Kornienko N. Electrochemical biomass valorization on gold-metal oxide nanoscale heterojunctions enables investigation of both catalyst and reaction dynamics with operando surface-enhanced Raman spectroscopy. Chem. Sci. 2020, 11 (7), 1798–1806. 10.1039/D0SC00136H. 32180924 PMC7053505

[ref35] Lu Y.; Liu T.; Dong C.-L.; Huang Y.-C.; Li Y.; Chen J.; Zou Y.; Wang S. Tuning the selective adsorption site of biomass on Co_3_O_4_ by Ir single atoms for electrosynthesis. Adv. Mater. 2021, 33 (8), 2007056 10.1002/adma.202007056. 33470476

[ref36] Wang Y.; Gao T.; Lu Y.; Wang Y.; Cao Q.; Fang W. Efficient hydrogenation of furfural to furfuryl alcohol by magnetically recoverable RuCo bimetallic catalyst. Green Energy Environ. 2022, 7 (2), 275–287. 10.1016/j.gee.2020.09.014.

[ref37] Zhang W.; Wang Y.; Gu B.; Tang Q.; Cao Q.-E.; Fang W. Regulating the interaction within Pd-Cu dual metal sites for selective hydrogenation of furfural using ambient H_2_ pressure. ACS Sustain. Chem. Eng. 2023, 11 (34), 12798–12808. 10.1021/acssuschemeng.3c03763.

[ref38] Magdić Košiček K.; Kvastek K.; Horvat-Radošević V. Hydrogen evolution on Pt and polyaniline modified Pt electrodes – a comparative electrochemical impedance spectroscopy study. J. Solid State Electrochem. 2016, 20 (11), 3003–3013. 10.1007/s10008-016-3246-z.

[ref39] Liu H.; Li W. Recent advances in paired electrolysis of biomass-derived compounds toward cogeneration of value-added chemicals and fuels. Curr. Opin. Electrochem. 2021, 30, 100795 10.1016/j.coelec.2021.100795.

[ref40] Cao X.; Wulan B.; Wang Y.; Ma J.; Hou S.; Zhang J. Atomic bismuth induced ensemble sites with indium towards highly efficient and stable electrocatalytic reduction of carbon dioxide. Sci. Bull. 2023, 68 (10), 1008–1016. 10.1016/j.scib.2023.04.026. 37169613

[ref41] Chen L.; Yu C.; Song X.; Dong J.; Mu J.; Qiu J. Integrated electrochemical and chemical system for ampere-level production of terephthalic acid alternatives and hydrogen. Nat. Commun. 2024, 15, 8072 10.1038/s41467-024-51937-y. 39277577 PMC11401954

[ref42] Gómez-Gallego M.; Sierra M. A. Kinetic isotope effects in the study of organometallic reaction mechanisms. Chem. Rev. 2011, 111 (8), 4857–4963. 10.1021/cr100436k. 21545118

[ref43] Lin Y.; Deng C.; Wu L.; Zhang Y.; Chen C.; Ma W.; Zhao J. Quantitative isotope measurements in heterogeneous photocatalysis and electrocatalysis. Energy Environ. Sci. 2020, 13 (9), 2602–2617. 10.1039/D0EE01790F.

[ref44] Yang Y.; Xu D.; Zhang B.; Xue Z.; Mu T. Substrate molecule adsorption energy: An activity descriptor for electrochemical oxidation of 5-Hydroxymethylfurfural (HMF). Chem. Eng. J. 2022, 433, 133842 10.1016/j.cej.2021.133842.

[ref45] Ge R.; Wang Y.; Li Z.; Xu M.; Xu S.-M.; Zhou H.; Ji K.; Chen F.; Zhou J.; Duan H. Selective electrooxidation of biomass-derived alcohols to aldehydes in a neutral medium: Promoted water dissociation over a nickel-oxide-supported ruthenium single-atom catalyst. Angew. Chem., Int. Ed. 2022, 61 (19), e202200211 10.1002/anie.202200211. 35170172

[ref46] Wang X.; Chen N.; Liu X.; Shi Y.; Ling C.; Zhang L. Ascorbate guided conversion of hydrogen peroxide to hydroxyl radical on goethite. Appl. Catal., B 2021, 282, 119558 10.1016/j.apcatb.2020.119558.

