# Supplementary material for: Pairing Electrocatalytic Reduction and Oxidation of Biomass-Derived 5-Hydroxymethylfurfural into Highly Value-Added Chemicals
Source: JACS Au. 2025 Jan 2;5(2):937–47. doi: 10.1021/jacsau.4c01135 (PMC11862940; doi:10.1021/jacsau.4c01135)
Supplement: Supplementary file 1 — au4c01135_si_001.pdf [file au4c01135_si_001.pdf]

Supporting Information

Pairing Electrocatalytic Reduction and Oxidation of Biomass-Derived 5-Hydroxymethylfurfural into Highly Value-Added Chemicals

Man Zhang,[ab] Zhikeng Zheng,[a] Xiaodie Zhang,[a] Zhiwei Jiang,[a] Xue Yong,[c] Ke Li,[a] Xin Tu,[c*] Kai Yan[a*]

[a] Guangdong Provincial Key Laboratory of Environmental Pollution Control and Remediation Technology, School of Environmental Science and Engineering, Sun Yat-sen University, Guangzhou 510275, China

[b] College of Chemistry and Environment, Southwest University for Nationalities, Chengdu 610207, China

[c] Department of Electrical Engineering and Electronics, University of Liverpool, Liverpool L69 3GJ, UK

* Email: xin.tu@liverpool.ac.uk (Xin Tu), yank9@mail.sysu.edu.cn (Kai Yan).

Experimental Section

Chemicals and Equipment

Platinum(II) acetylacetonate (Pt(acac)$_2$, 97%) and ruthenium(III) acetylacetonate (Ru(acac)$_3$, 97%) were purchased from Macklin. Potassium phosphate monobasic (KH_2PO_4, 99.5%), 5-hydroxymethylfurfural (HMF, 98.6%), 2,5-dihydroxymethylfuran (DHMF, 98%) and 2,5-furandicarboxylic acid (FDCA, 98%) were purchased from Aladdlin and used without further purification. Potassium hydroxide (KOH, 95%), furfural (FF, 98%) and furfuryl alcohol (FA, 99%) were purchased from Shanghai Macklin Biochemical Co., Ltd. Ethanol (C_2H_6OH, 99%) was from Guangdong Guanghua Biochemical Co., Ltd and PBS (0.01 M/0.1 M) purchased by Biosharp. The nitric acid (HNO_3, 68%) and sulfuric acid (H_2SO_4, 95-98%) were manufactured by Guangzhou Chemical Reagent Factory. High purity argon (≥ 99.999%) and oxygen (≥ 99.999%) were purchased from Dacheng (Guangzhou) Gas Co., Ltd. All reagents were raw and used to the experiment. Ultra-pure water (~18.25 MΩ×cm) was obtained by the reverse osmosis (RO) technology and ultra-purification system filtration (Ulupure, China) using for the whole experiment. The pH meter (Starter 3100) with a resolution ratio of 0.01 pH was purchased from OHAUS Laboratory (USA).

Characterization

Scanning electron microscopy (SEM) images were obtained by a Gemini500 scanning electron microscope operating at 15 kV with the distinguishability of 0.6 nm. Energy dispersive X-ray spectroscopy (EDX) mapping was performed on the catalysts equipped with an Aztec Xmax 50 EDS detector at 15 kV. The transmission electron microscopy (TEM) and high-resolution TEM (HRTEM) images were taken on a FEI Tecnai G^2 F30 instrument operated at 200 kV to survey the material microstructure and lattice fringes. X-ray diffraction (XRD) patterns were collected on electrode X-ray diffractometer at 40 kV and 40 mA using Co-Kα radiation (Rigaku Ultima IV, Janpan) and the scanning rage of theta (2θ) was from 5° to 70° at a scan rate of 2° min^{-1}. Thermo ESCALab250 X-ray photoelectron spectrometer (USA) was used to evaluate X-ray photoelectron spectroscopy (XPS) experiments, which used a monochromatic Al K as the excitation source at 150 W and pass energy of 20 eV.

Analysis of Products

To quantitatively analyze reactant of electrocatalytic reaction of HMF, the sample was collected and diluted with water and then analyzed by the high-performance liquid chromatography (HPLC, Shimadzu Prominence LC-20A) equipped with a variable wavelength detector. HMF and its reductive products were determined using the Shimadzu C 18 column (4.6 mm × 150 mm, 5 μm). The column was operated at 45 °C with a binary gradient pumping method of water and acetonitrile at a 0.6 mL min^{-1} flow rate. The acetonitrile fraction was increased from the initial 15% (v/v) to 30% over the 5 to 8.33 min period, then was increased from 30% to 60% over the 8.33 to 10 min period, and then was decreased to 15% from the 10 to 13 min period and kept the ratio until 20 min. DHMF and HMF eluted around 6.0 and 7.1 minutes, respectively. The conversion and selectivity are calculated as follows:

Equation S1: $\text{Conversion (\%)} = (1 - \frac{n'_{\text{substrate}}}{n_{\text{substrate}}}) \times 100\ \%.$

Equation S2: $\text{Selectivity (\%)} = \frac{n_{\text{product}}}{n_{\text{substrate}}} \times 100\ \%.$

Equation S3: $\text{FE (\%)} = \frac{n_{\text{product}}}{\text{Total charge passed } (C)/(F\times n)} \times\ 100\ \%.$

Where F is the Faraday constant (96485 C mol^{-1}) and n is the number of electrons required for the conversion of the HMF to DHMF.

Pairing Reactions

The electrolytes used included 1 M potassium hydroxide (KOH) solution (pH 13.6), 0.5 M H_2SO_4 (pH 0.38) and 0.01 M PBS (pH 7.4). Then, 30 mL of electrolyte was added to both sides of an H-type cell (50 mL total volume) with a Nafion 117 membrane. The membrane was treated with 3% hydrogen peroxide and 0.5 M H_2SO_4. As in previous works, electrochemical testing was conducted using an Autloab M204 (Metrohm) in a typical three-electrode system. The working electrode was PtRu alloy on carbon fiber paper, while a Pt wire (Φ = 1 mm, 4 cm in length, Shanghai CH Instruments Ins.) was used as the counter electrode for all electrochemical experiments. The reference electrode was Ag|AgCl (Gaoss Union, Single salt bridge), used in PBS electrolytes. The acquired potential was standardized to the RHE according to the equation (Equation S4: $E_{RHE} = E^{\ominus}_{(Ag|AgCl)} + 0.0591 \times pH + 0.197$). The linear sweep voltammetry (LSV) curves were recorded in the potential range of 0.2 to -0.9 V_{RHE} at a scan rate of 10 mV s^{-1}. The pairing

reaction of the electrochemical reduction and oxidation was carried out at a constant potential/current as shown in Scheme S1. The cathode electrode was PtRu alloy on carbon fiber paper in 0.1 M PBS electrolyte, while the anode was a $NiCo(OOH)_x$ nanosheet electrode in 1 M KOH electrolyte. For all electrochemical tests, the IR value was compensated to account for the voltage drop between the working electrode and reference electrode using Nova Software. No IR compensation was applied to the pairing reaction tests. Electrochemical impedance spectroscopy (EIS) was performed by applying an AC voltage with a 5 mV amplitude in a frequency range from 100000 to 0.1 Hz, recorded at different potential for electrooxidation. All experiments were conducted at 25 ℃ and 101.95 kPa.

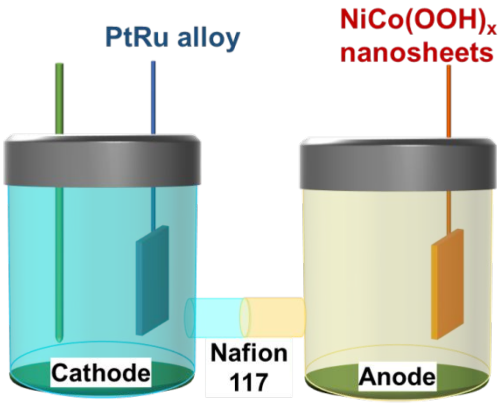

Scheme S1. The cell device for the paired reaction, featuring both cathode and anode electrodes.

Analytical Methods of Cumulative [OH*]

Quantification of OH* was carried out using benzyl alcohol (BA) as a probe molecule. The reaction products of OH* and BA are three hydroxybenzoic acid isomers, including ortho- hydroxybenzoic acid (o-HBA), meta-hydroxybenzoic acid (m-HBA), and para-hydroxybenzoic acid (p-HBA). The mole ratio of o-HBA, m-HBA and p-HBA was 1.7 : 2.3 : 1.2. Among the three isomers, p-HBA could be easily determined by high performance liquid chromatography (HPLC, Shimadzu Prominence LC-20A) with a Shimadzu C 18 column (4.6 mm × 150 mm, 5 μm). As reported previously [1], the concentration of p-HBA could be used to quantify the concentration of cumulative OH* according to Equation of Cumulative [OH*] = [p-HBA] × 5.87. The injection volume was 10 μL. The mobile phase was composed of acetonitrile and 0.2% acetic acid aqueous solution (30:70, v/v). The flow rate was 1 mL min^{-1}, and the detection wavelength was

270 nm.

H/D Isotope Analysis

H/D isotope substitution tests were performed to reveal the kinetic isotope effects (KIE) of electrochemical reduction/oxidation reactions. KIE is the change in reaction rate when an atom in a reactant is replaced by its isotope. If such a change in the reactants does not offset the change in the transition state. In other words, if the ground state vibration energy difference between the reactants and the transition state before and after isotope substitution is different, the reaction rate will change according to the transition state theory. The formula is as follows: $k = \frac{k_B T}{h} \frac{Q^{\ddagger}}{Q_A Q_B} exp\left(-\frac{\Delta\varepsilon_0}{k_B T}\right)$

Where k is the rate constant. k_B is Boltzmann's constant. T is temperature. $Q^{\ddagger}$, Q_A, Q_B are partition functions of the transition state, reactant A, and reactant B, respectively. $\Delta\varepsilon_0$ is the difference in ground state vibration energy between the reactant and the transition state. The reaction rate ratio before and after isotope substitution is called KIE.

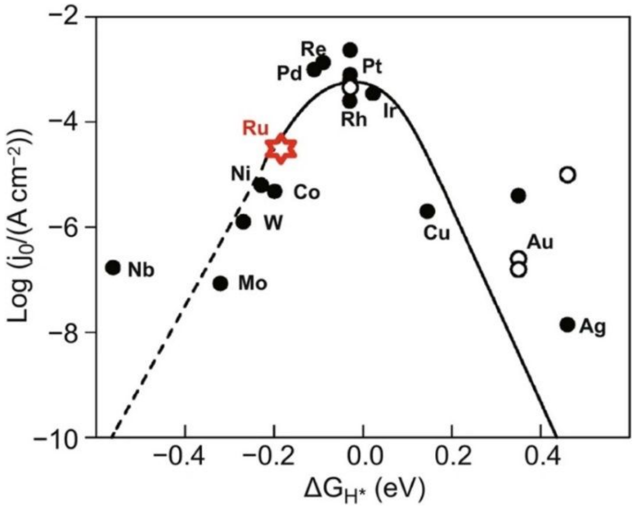

Figure S1. Volcano plot of hydrogen adsorption free energy (ΔG_{H*}) of different metal catalysts in HER. With the exception of Ru, the data for the other metals were from the summary of Nørskov et al. High H coverage of metals on the left side of the volcano (1 monolayer (ML)), lower on the right side (0.25 ML). The dashed line indicates that the metals which bind H stronger than 0.2 eV H^{-1} usually form oxides at U = 0 V. The open circles are (111) data, whereas the filled circles are polycrystalline [2]. The data of Ru are from Hoster et al., corresponding to the calculation data of Ru (0001) at 1.1 ML [3]. Reproduced under terms of the CC-BY license [4]. Copyright 2021, published by Springer Nature.

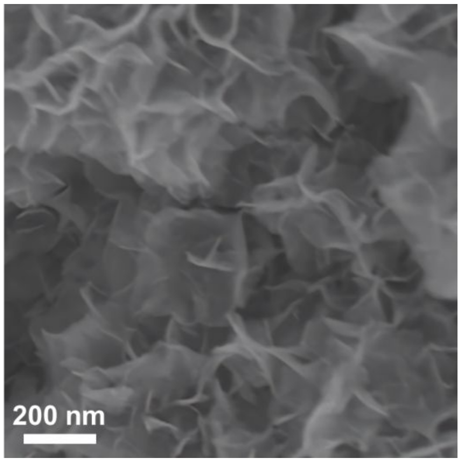

Figure S2. SEM image of $NiCo(OOH)_x$ nanosheets.

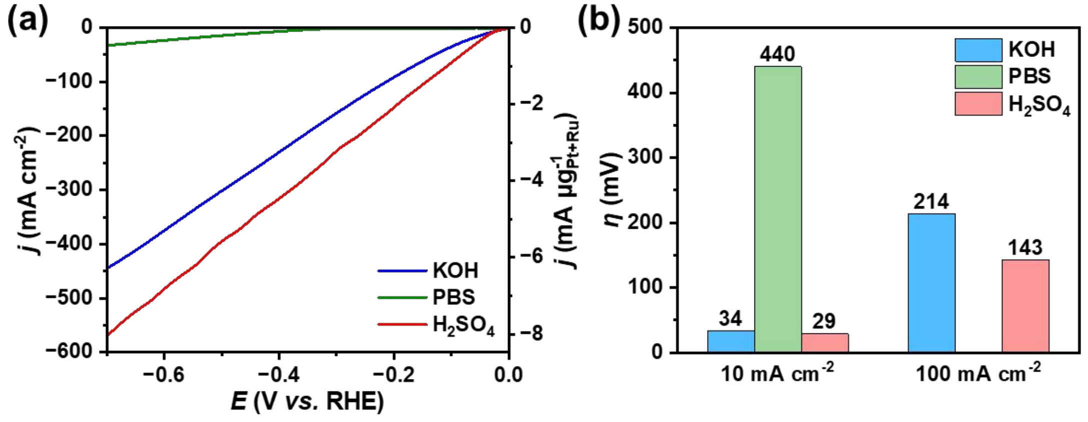

Figure S3. (a) LSV curves and (b) overpotential in KOH, PBS, H_2SO_4 electrolyte.

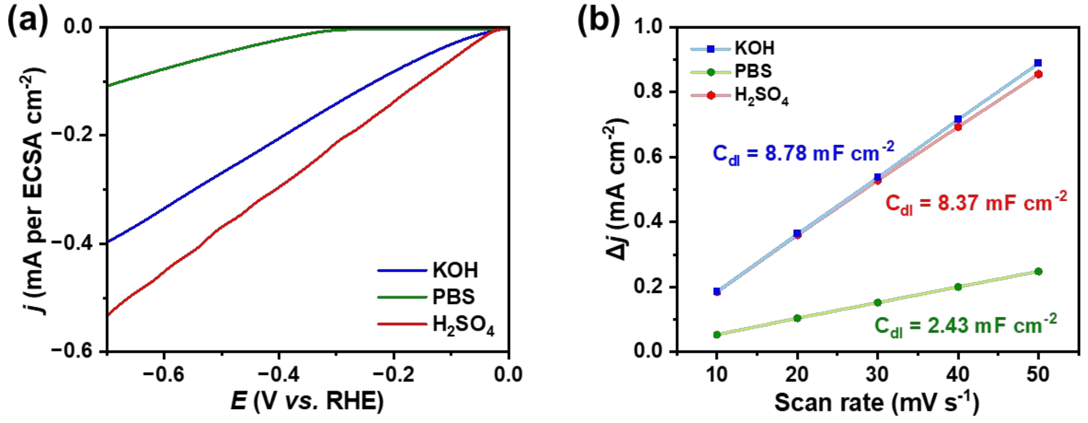

Figure S4. (a) ECSA-normalized LSV curves and (b) ECSA calculated in KOH, PBS, H_2SO_4 electrolyte.

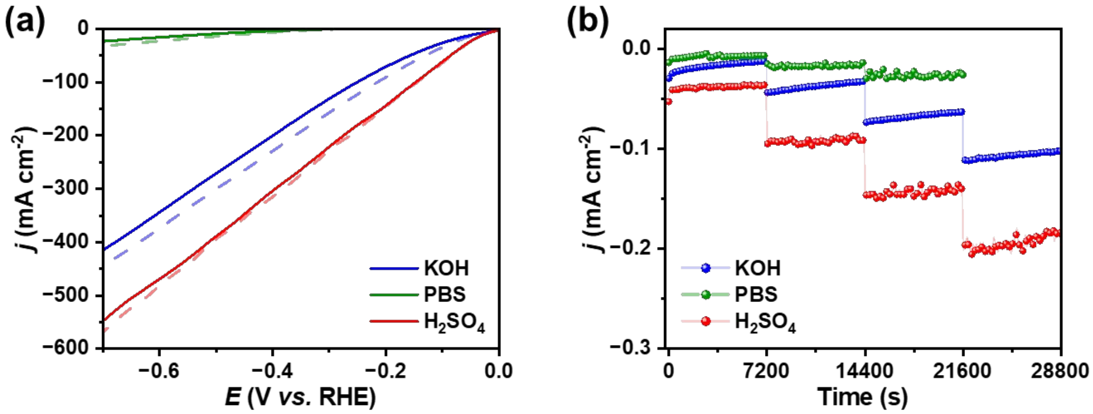

Figure S5. The durability of the PtRu alloy in KOH, PBS, H_2SO_4 electrolyte, including LSV curves of before and after 1000 cycles, and potentiostatic tests.

LSV curves and C_{dl} values were performed to assess the performance of the PtRu alloy in various electrolytes. Figure S3a depicts the LSV curves of the PtRu alloy in 1 M KOH, 0.5 M H_2SO_4, and 0.1 M PBS, indicating superior electrochemical performance in 0.5 M H_2SO_4. Figure S3b shows that the PtRu alloy catalyst at 0.5 M H_2SO_4 has the lowest overpotential (29 mV at 10 mA cm^{-2} and 143 mV at 100 mA cm^{-2}) compared with the other two electrolytes. C_{dl} values were obtained by cyclic voltammetry (CV) cycles testing at scan rates from 10 to 50 mV s^{-1} to evaluate the electrochemical surface area. The calculated C_{dl} value for the PtRu alloy in 1 M KOH is higher than in other electrolytes, measuring 8.78 mF cm^{-2} (Figure S4). However, upon normalizing the current density with respect to the electrochemical active surface area, the alloy exhibits optimal electrochemical activity in acidic medium. After undergoing 1000 cycles of CV in various electrolytes, the PtRu alloy demonstrates remarkable cycling stability, maintaining nearly constant current density. The potentiostatic tests also indicate that the PtRu alloy maintains long-term durability at various potentials, as shown in Figure S5.

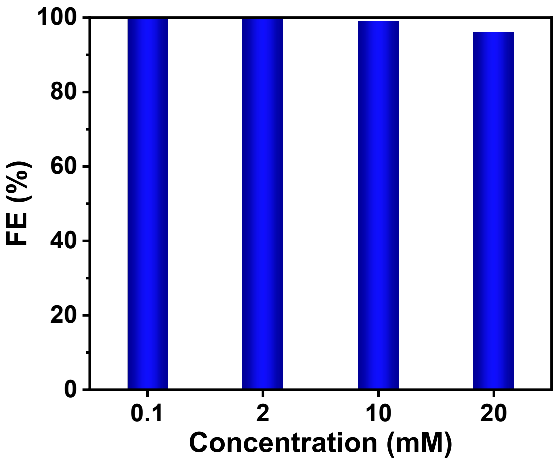

Figure S6. The FE in 0.1, 2, 10, 20 mM HMF electrolyte on the PtRu alloy.

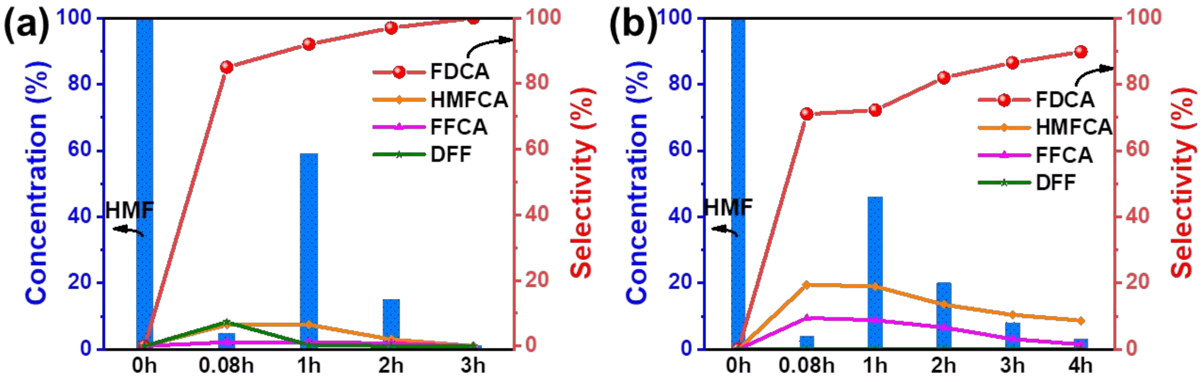

Figure S7. HMF oxidation at anode. (a) HMF concentration and products selectivity in KOH using H_2O for PERO. (b) HMF concentration and products selectivity in KOH using D_2O for PERO.

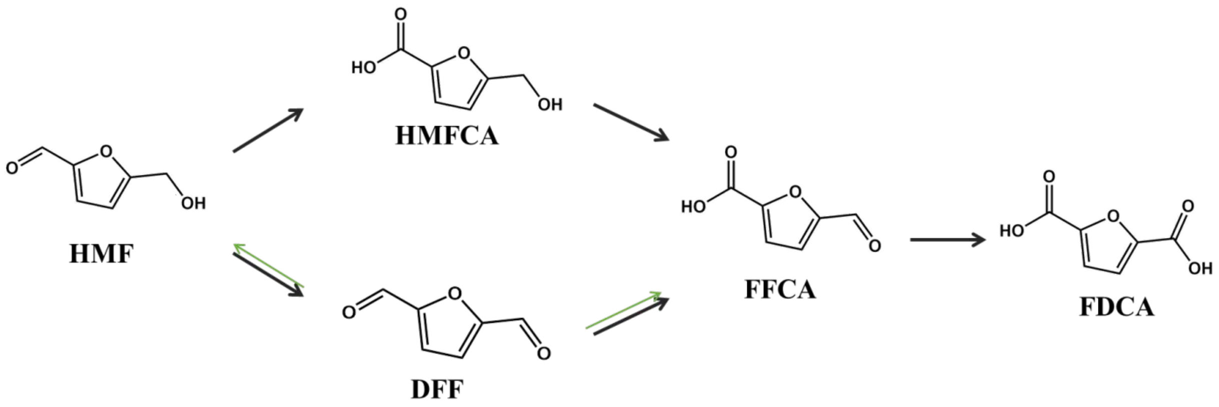

Figure S8. Two possible electrooxidation pathways for the synthesis of FDCA from HMF. Note: the non-electrochemical pathway (green) is included.

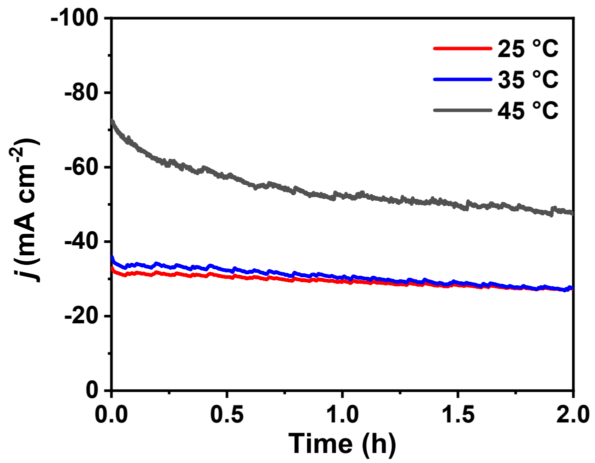

Figure S9. Potentiostatic tests of the PtRu alloy under 25, 35 and 45 ℃.

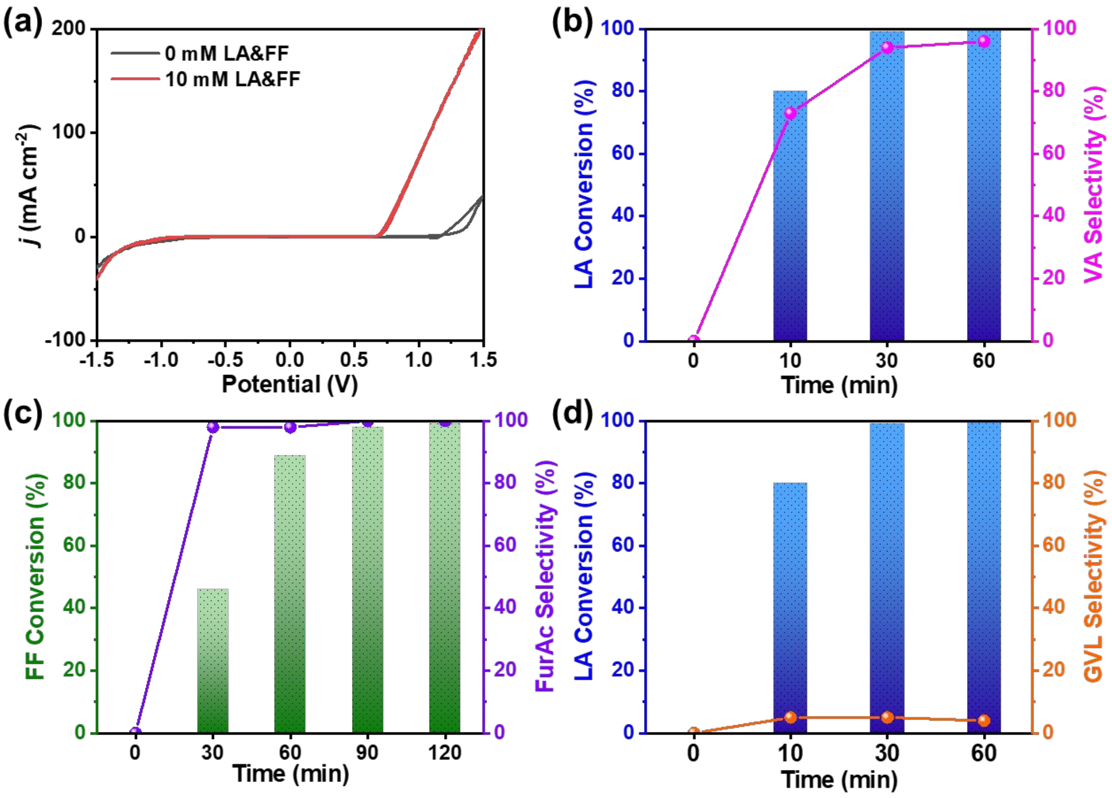

Figure S10. LA reduction at cathode and FF oxidation at anode. (a) CV curves after adding LA at cathode and FF at anode. (b) LA conversion and VA selectivity. (c) FF conversion and FurAc selectivity. (d) LA conversion and GVL selectivity.

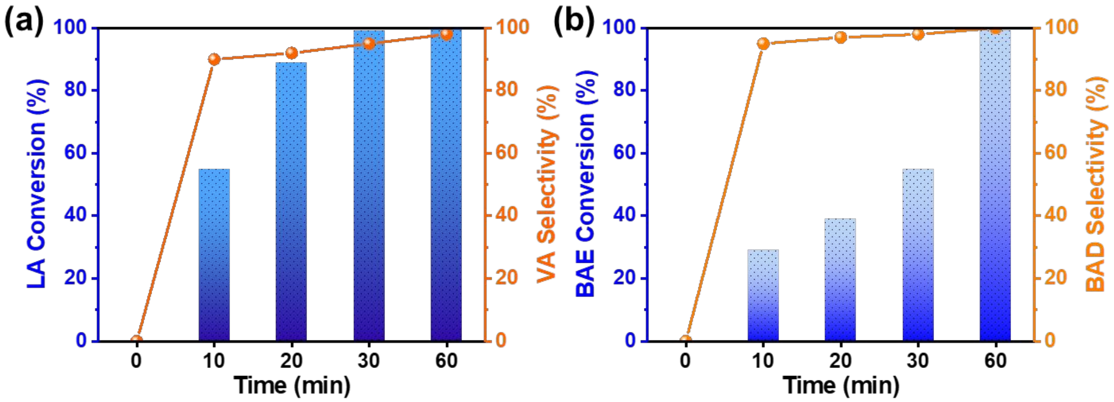

Figure S11. LA reduction at cathode and BAE oxidation at anode. (a) LA conversion and VA selectivity. (b) BAE conversion and BAD selectivity.

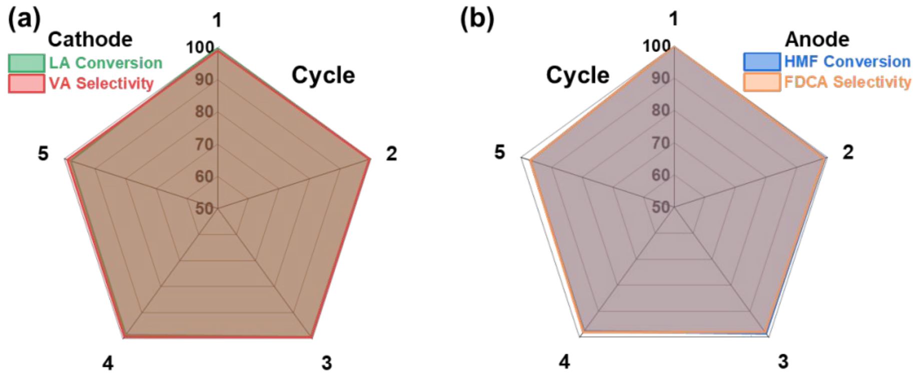

Figure S12. The catalytic performance for five cycles of LA reduction || HMF oxidation system.

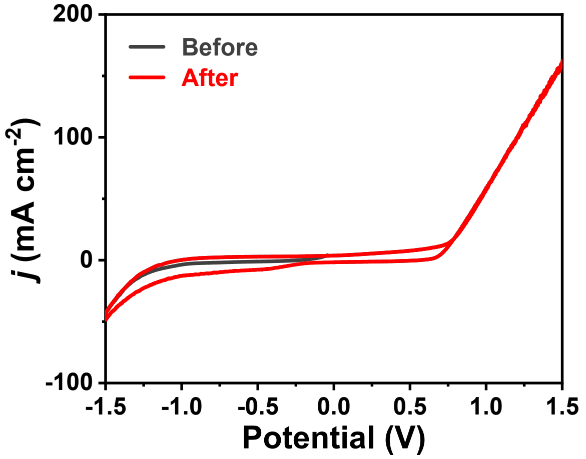

Figure S13. CV curves in the potential range of -1.5 to 1.5 V before and after five cycles in the LA reduction || HMF oxidation system.

Table S1. The comparison of pairing electrocatalytic reduction and oxidation performance of biomass-derived platform chemicals into highly value-added chemicals.

**Cathode**				**Anode**				**Ref.**
Substrate	Product	Conversion (%)	Selectivity (%)	Substrate	Product	Conversion (%)	Selectivity (%)	
m-NP	m-Ap	≥99	≥99	FF	FurAc	≥99	≥99	5
p-NP	p-AP	≥99	≥99	HMF	FDCA	≥99	≥99	5
HMF	DHMF	19.7	89.3 (FE)	HMF	FDCA	100	96 (FE)	6
HMF	DHMF	88	77	/	/	/	/	7
HMF	DHMF	29	28	/	/	/	/	8
HMF	DHMF	87.3	85.1	/	/	/	/	9
HMF	DHMF	98	94	HMF	FDCA	99	99	This work
LA	VA	100	100	HMF	FDCA	100	99	
LA	VA	100	100	FF	FurAc	100	99	
LA	VA	100	>99	BAE	BAD	100	>99	

References

(1) Chen, N.; Huang, Y.; Hou, X.; Ai, Z.; Zhang, L. Photochemistry of hydrochar: Reactive oxygen species generation and sulfadimidine degradation. *Environ. Sci. Technol.* **2017**, *51* (19), 11278-11287.

(2) Skúlason, E.; Tripkovic, V.; Björketun, M. E.; Gudmundsdóttir, S.; Karlberg, G.; Rossmeisl, J.; Bligaard, T.; Jónsson, H.; Nørskov, J. K. Modeling the electrochemical hydrogen oxidation and evolution reactions on the basis of density functional theory calculations. *J. Phys. Chem. C* **2010**, *114* (42), 18182-18197.

(3) Hoster, H. E. Anodic hydrogen oxidation at bare and Pt-modified Ru(0001) in flowing electrolyte–theory versus experiment. *MRS Online Proc. Libr.* **2011**, *1388* (1), 10.

(4) Yang, Y.; Yu, Y.; Li, J.; Chen, Q.; Du, Y.; Rao, P.; Li, R.; Jia, C.; Kang, Z.; Deng, P.; et al. Engineering ruthenium-based electrocatalysts for effective hydrogen evolution reaction. *Nano-Micro Lett.* **2021**, *13* (1), 160.

(5) Zhang, P.; Sheng, X.; Chen, X.; Fang, Z.; Jiang, J.; Wang, M.; Li, F.; Fan, L.; Ren, Y.; Zhang, B.; et al. Paired electrocatalytic oxygenation and hydrogenation of organic substrates with water as the oxygen and hydrogen source. *Angew. Chem. Int. Ed.* **2019**, *58* (27), 9155-9159.

(6) Chadderdon, X. H.; Chadderdon, D. J.; Pfennig, T.; Shanks, B. H.; Li, W. Paired electrocatalytic hydrogenation and oxidation of 5-(hydroxymethyl)furfural for efficient production of biomass-derived monomers. *Green Chem.* **2019**, *21* (22), 6210-6219.

(7) Piao, G.; Yoon, S. H.; Cha, H. G.; Han, D. S.; Park, H. Porous dendritic BiSn electrocatalysts for hydrogenation of 5-hydroxymethylfurfural. *J. Mater. Chem. A* **2022**, *10*, 24006.

(8) Zhang, L.; Zhang, F.; Michel Jr, F. C.; Co, A. C. Efficient electrochemical hydrogenation of 5-hydroxymethylfurfural to 2,5-bis(hydroxymethyl)furan on Ag-displaced nanotextured Cu catalysts. *ChemElectroChem* **2019**, *6* (18), 4739-4749.

(9) Ji, K.; Xu, M.; Xu, S.; Wang, Y.; Ge, R.; Hu, X.; Sun, X.; Duan, H. Electrocatalytic hydrogenation of 5-hydroxymethylfurfural promoted by a Ru_1Cu single-atom alloy catalyst. *Angew. Chem. Int. Ed.* **2022**, *61* (37), e202209849.
